# Cell-mediated and cell membrane-coated nanoparticles for drug delivery and cancer therapy

**DOI:** 10.20517/cdr.2020.55

**Published:** 2020-11-03

**Authors:** Serkan Yaman, Uday Chintapula, Edgar Rodriguez, Harish Ramachandramoorthy, Kytai T. Nguyen

**Affiliations:** 1 Department of Bioengineering, University of Texas at Arlington, Arlington, TX 76010, USA.; 2 Joint Bioengineering Program, University of Texas Southwestern Medical Center, Dallas, TX 75235, USA.

**Keywords:** Cell membrane-based drug delivery, cell-mediated drug delivery, membrane receptors, drug carriers, cancer drug resistance, nanoparticles

## Abstract

Nanotechnology-based drug delivery platforms have been developed over the last two decades because of their favorable features in terms of improved drug bioavailability and stability. Despite recent advancement in nanotechnology platforms, this approach still falls short to meet the complexity of biological systems and diseases, such as avoiding systemic side effects, manipulating biological interactions and overcoming drug resistance, which hinders the therapeutic outcomes of the NP-based drug delivery systems. To address these issues, various strategies have been developed including the use of engineered cells and/or cell membrane-coated nanocarriers. Cell membrane receptor profiles and characteristics are vital in performing therapeutic functions, targeting, and homing of either engineered cells or cell membrane-coated nanocarriers to the sites of interest. In this context, we comprehensively discuss various cell- and cell membrane-based drug delivery approaches towards cancer therapy, the therapeutic potential of these strategies, and the limitations associated with engineered cells as drug carriers and cell membrane-associated drug nanocarriers. Finally, we review various cell types and cell membrane receptors for their potential in targeting, immunomodulation and overcoming drug resistance in cancer.

## INTRODUCTION

Based on recent trends, cancer is expected to become the leading cause of death in the world and effective treatment strategies are needed to address the increasing trend in incidents of cancer^[1]^. Chemotherapy has been established as a standard treatment in various cancer therapies with hundreds of antitumor drugs developed and approved for human use. However, drug sensitivity in cancer cells has been reduced due to the emergence of multiple drug resistance (MDR) induced by various factors including ATP-dependent drug efflux out of the cell, selective stress of drugs, altered DNA repair mechanisms, cellular heterogeneity in the tumors, and other drug barriers^[2]^. Several nanotechnology-based drug delivery systems utilize biomaterials including polymers, metals and lipids to improve the therapeutic index by improving drug loading efficacy, controlling drug release kinetics to address the challenges associated with free drugs such as systemic side effects, pharmacokinetic diversity, and physiological variety, as well as achieving ideal dose regimen and other antagonistic effects of combinatorial drug therapies used in addressing MDR. But the efficient bioavailability with those platforms has not yet been achieved due to their relatively simple structure compared to complex biomolecule interactive systems *in vivo* and they still suffer from the “foreign-body” aspects, leading to side effects, immune clearance and poor targeting abilities due to the protein corona formation *in vivo*, making them unable to meet clinical expectations^[3]^.

Nanoparticles (NPs) as a carrier loaded with drugs can be directed towards a specific target by the use of various surface moieties involved in the complex biological mechanisms^[4]^. A bottom-up approach of surface functionalization is commonly adopted in the preparation of NPs for targeted drug delivery. Surface functionalization incorporates functional moieties such as antibodies, enzymes, ligands, and other functional target molecules onto the surface of drug delivery carriers via various chemical and non-chemical interactions. Though NPs have shown optimal therapeutic results, they still lack in multifunctional applications such as improved circulation, targeting, homing, immunomodulation, and/or in combination. Although more than 80% of NP systems published are designed to treat cancer through their enhanced permeability and retention (EPR) effects, only a few tumors have been reported to achieve NP accumulation through EPR effects^[5,6]^. Various cells, due to their innate features, can reach tumor sites via their membrane components which may help in reducing toxicities arising from the current one-size-fits-all approach. Patient-to-patient *in vivo* biology varies in immune responsiveness and disease pathology; for example, tumor heterogeneity in cancers requires a bio-interfacing approach to address limitations of synthetic NP drug delivery systems. Together, the challenges faced in NP drug carriers demands more efficient and safer approaches to achieve therapeutic potential and to meet clinical expectations.

**Figure 1.**
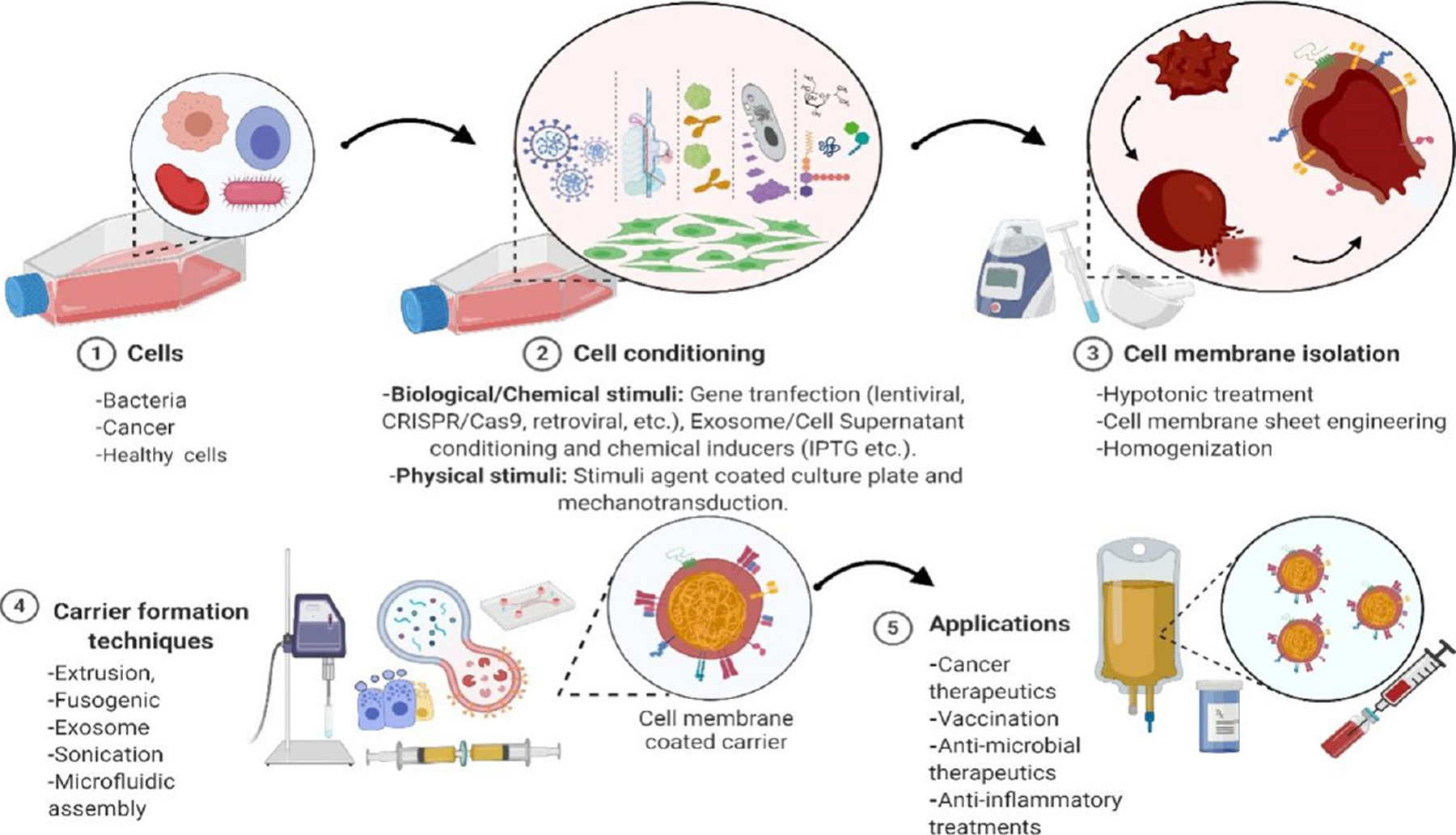
Preparation of cell/cell membrane-based payload delivery and its applications. CRISPR: clustered regularly interspaced short palindromic repeats; Cas9: CRISPR associated protein 9; IPTG: isopropyl β-D-1-thiogalactopyranoside

Cell- and cell membrane-based NPs possess multifunctional abilities, which make them ideal in NP-based cancer therapies. Cell membrane-coated NP (CMCNPs) have been increasingly studied for their mimicry of cell surface functionality, which can aid in reducing the immune responses of synthetic NPs *in vivo* and introduce the ability to combine both natural and synthetic materials concisely as shown in Figure 1. Cell- and cell membrane-based drug carriers exhibiting intrinsic properties of *in vivo* biology have been shown to overcome the challenges faced by synthetic NP-based drug carriers and achieve acceptable toxicity and better biocompatibility than their synthetic counterparts^[7–12]^. Major advantages of using cell- and cell membrane-based drug carriers include provision of immune escape and specific tumor targeting imparted by the cell membrane proteins leading to improved EPR in cancer therapies, and an ability to generate desired cytotoxic immunomodulatory effects via cell surface engineering, leading to tumor regression^[13–18]^.

**Figure 2.**
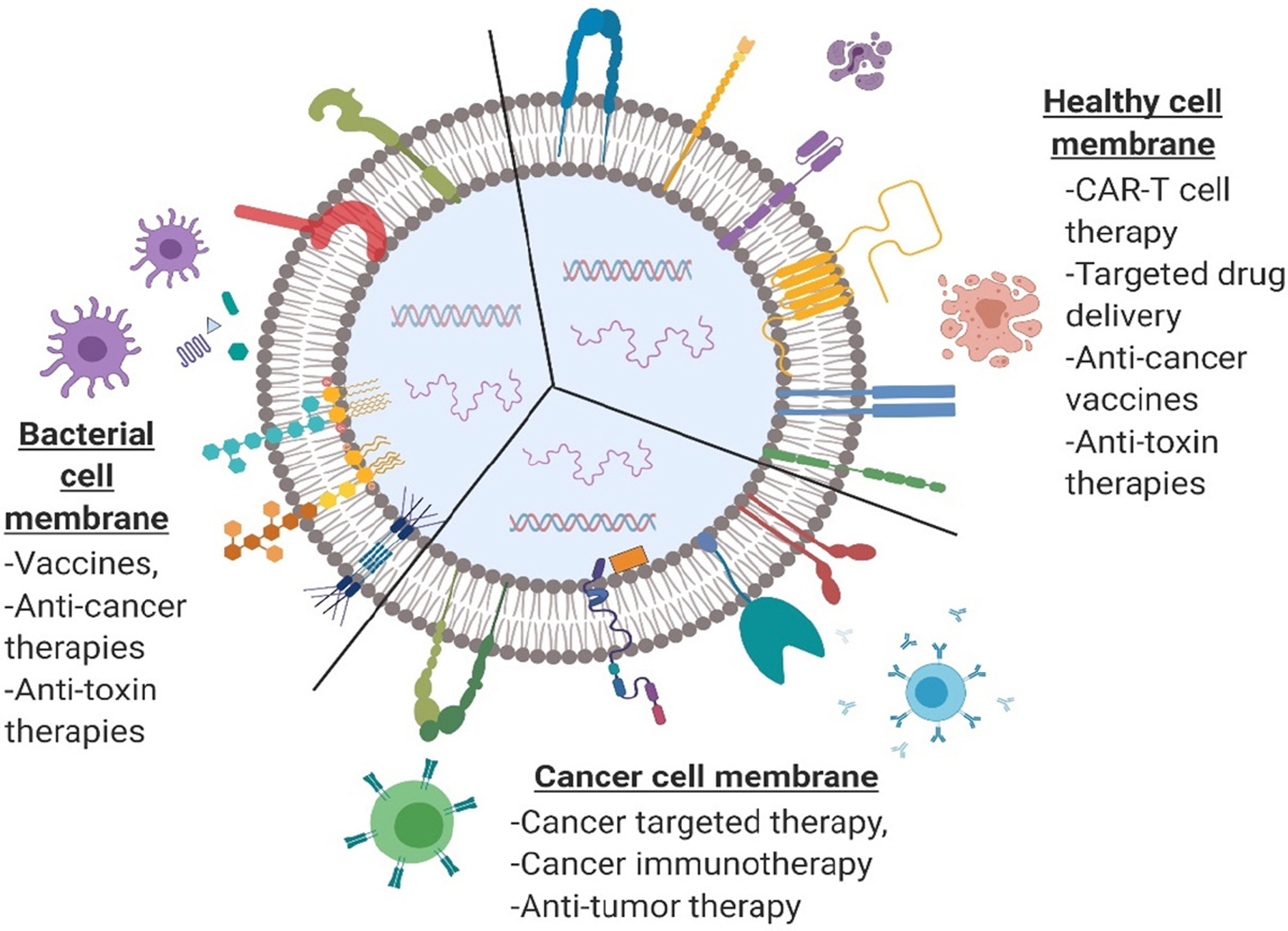
Illustration showing major cell types and the use of cell membranes in drug delivery, immunotherapy, and immunomodulation. CAR: chimeric antigenic receptor

Cancer is a complex disease involving various cells and their membrane interactions in the tumor microenvironment, such as immune suppression via PD-1/PD-L1 axis in T cells, recruitment of stem cells via membrane receptor-mediated CXCR4/CXCL2 chemokine axis, maturation of immune cells via membrane interactions, and various other chemical interactions, which uncover the potential of using cells in cell- and cell membrane-based drug delivery. Recently, discovered mechanisms of these interactions are being explored by NP technology to develop various treatment strategies^[19–21]^. Efficient navigation, while maintaining the integrity of the drug carrier and physiologically pertinent interactions within complex biological environments, can be achieved using cell membrane-coated NPs with a relatively higher circulation-time^[22]^. Accordingly, researchers in NP-based drug delivery have shifted their focus to the use of engineering cells and/or cell-derived sources for cancer therapies and immunomodulation, as shown in Figure 2. Bioengineered components including whole cells, cell membranes, and exosomes are being employed in anticancer or immunomodulating drugs^[23]^ and vaccine-loaded carriers^[24,25]^. Cell- and/or cell membrane-based NP drug delivery platforms can be applied in numerous ways to alter biological functions and pathways and be used in targeting and manipulating their destination to achieve desired therapeutic responses. Therefore, it is of critical importance to understand the cell membrane mechanisms involved in targeting, altering immune responses, and eliciting therapeutic outcomes. Along with recent cell- and cell membrane-based drug carriers in cancer and immunomodulation, we provide an up-to-date review of current and potential cell types and cell membrane receptors involved in cancer therapy and immunomodulation.

## CELLS AS DRUG CARRIERS AND LIMITATIONS OF WHOLE CELLS AS CARRIERS

As cell-based drug delivery systems involve the utilization of the cell’s biological features for the development of drug carriers, it is essential to understand their mechanisms *in vivo.* Multicellular organisms perform complex biological interactions between the host cell and the pathogen or diseased cells, which usually lead to highly acidic conditions inside the cells, dysregulated proliferation, release of inflammatory molecules, and other abnormalities. Most of the regulatory molecular interactions occurring in the immune system are in response to the abnormalities existing in diseases including cancer, autoimmune diseases, and infections. Recent breakthrough discoveries in underlying interactions in immunobiology and cancer biology have led to immune cell-based therapies as a new therapeutic approach in the clinic, especially in hematological cancers^[19–21,26]^.

Immune cells are extensively employed in cell-based drug carriers because of their ability to reach inflamed sites typically seen in many diseases, including cancer. In the last decade, engineered lymphoid cells were developed with various approaches to recognize specific disease antigens via their naïve receptors called “T cell receptors” (TCRs) or via novel engineered synthetic receptors called “chimeric antigenic receptors” (CARs). Novel approaches like the use of engineered T cells to give synthetic NPs a hitching ride to their destination show promising results in targeted drug delivery. Although there have been great advances in directing T cells to the tumor environment, immunosuppression in the tumor microenvironment still remains challenging^[27]^. T cells engineered using Synthetic Notch (SynNotch) circuits, when in contact with a specific antigen, promote cleavage of the transmembrane SynNotch receptor and release of transcriptional domain attached intracellularly, leading to entry into the nucleus followed by activation of targeted gene expression^[28]^. Cellular engineering strategies like these can be used to overcome challenges such as tumor immunosuppression and autoimmune activation^[29]^. In addition, they can successfully deliver molecules of interest at a pathologically-specific location, circumventing issues such as dilution of drug molecules and low circulation half-life faced in the direct injection of NP delivery^[30]^. Another similar strategy includes nuclease-deficient synthetic receptors (dCas9-synR), which accommodate combinatorial inputs such as proteins, lipids or sugars as an intracellular signal transduction module of antigen/antibody specific-cellular responses^[31]^.

**Figure 3.**
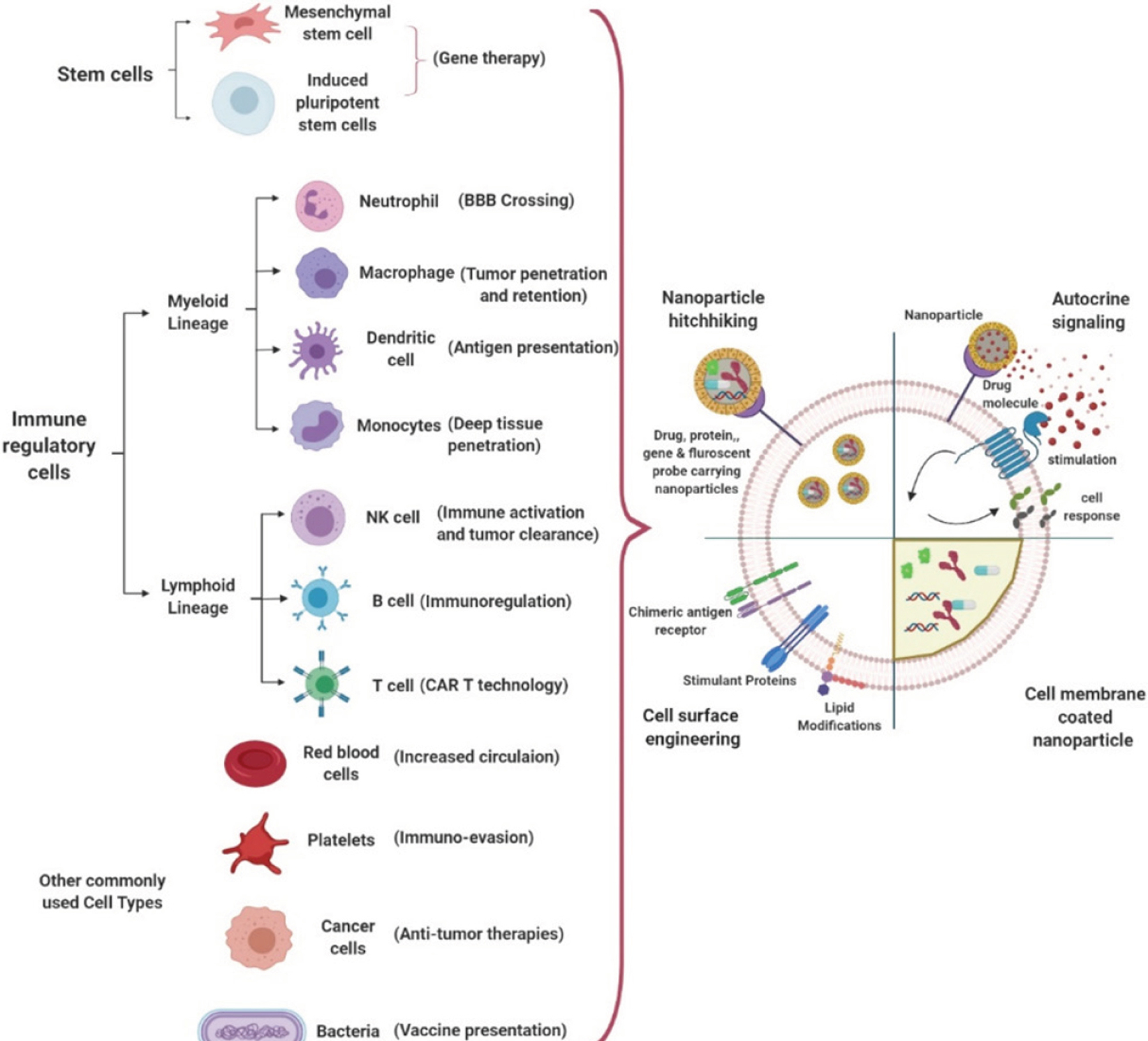
Current and potential cell types used for design of nanoparticle-based drug delivery in cancer therapy and immunomodulation along with their strategies to improve drug delivery (strategies include nanoparticle hitchhiking, autocrine signaling via cell membrane-bound nanoparticles, cell surface engineering and cell membrane-coated nanoparticle-based drug delivery). BBB: blood brain barrier

Cellular engineering techniques also have potential in designing novel signaling pathway circuits to improve cell differentiation where required, to provide the supplement antibody expression for regulatory feedback mechanisms, and to release immunosuppressive factors observed in autoimmune diseases, opening new avenues in immunomodulation. Some other drug delivery strategies employ cells as a carrier for targeted delivery of immunomodulatory drug-loaded NPs. In addition to immunosuppressive factors, the tumor microenvironment (TM) is hard to reach and plays a critical role for drug resistance development within the tumors^[32,33]^. Addressing these issues through applied therapies such as cell-based nanocarriers may help to overcome cancer drug resistance. For instance, targeting CTLA-4- and PD-1-expressing lymphocytes has shown to be a potential therapeutic approach towards tumor regression^[16]^. Conjugating immunomodulatory drug-loaded NPs on T cell surfaces (such as R848-toll-like receptor agonist or SD-208-inhibitor of TGFβ kinase) creates advantages such as more active seeking of cancer cells compared to only ligand-conjugated NPs, which have a reduced circulation half-life and are easily cleared from the system before reaching their targets^[16]^. In addition, immunomodulatory drugs loaded into NPs can induce checkpoint blockade and inhibit immunosuppressive mediators via autocrine and paracrine routes, leading to infiltration of CD8+ T cells into tumors. Cell-based drug delivery systems are beneficial over functionalized, synthetic drug carriers for many reasons including augmented with their intrinsic properties of reduced immunogenicity, imposed innate targeting ability, and improved circulation half-life. In addition to providing customizable signal pathways, cells provide an ideal platform to hitchhike drug carriers to desired locations [Figure 3].

Although they have advantageous biological intrinsic factors over synthetic nanocarriers, cells are highly sensitive to external manipulation including loading drug carriers, genetic and other morphological changes. Attaching or encapsulating drug-loaded NPs to the cell may present toxicity and alter its innate physiological abilities in maintaining homeostasis. It has been reported that RBC stability and plasticity have been affected due to chemical modifications of cell membranes. For instance, glycophorin A expression patterns have changed after crosslinking of NPs on the cell surface^[34]^. This change may lead RBCs to release damage-associated patterns (DAMP) and result in immune activation or opsonization by humoral elements^[35]^. In addition, abnormal release of internal proteins from modified cells may cause immune reactions such as hemoglobin-mediated immune responses^[36]^. The use of bacterial cell-mediated drug delivery may induce unfavorable immune responses in immune compromised terminally ill cancer patients^[37]^. Mesenchymal stem cell (MSC)-mediated drug delivery is employed to deliver payloads to tumor regions. However, it is reported that MSCs might contribute to tumor metastasis by releasing chemokines, helping tumors to suppress the immune system, to the differentiation of epithelial cells to become cancer-associated fibroblasts, and to the retention of the stemness of cancer cells^[38]^. Aspects of cell-based drug delivery require a thorough consideration of cell type selection and a combinatorial therapeutic approach. Limitations of cell-based drug delivery also include the limited surface availability and the cell’s own vital interactions with the environment. In addition, maintaining the released drug concentration in the therapeutic window while avoiding a cytotoxic effect to its cell carrier can make drug dosage optimization very challenging to achieve in cell-based therapies^[39]^.

## ADVANTAGES AND APPLICATIONS OF CELL MEMBRANE-COATED NANOPARTICLE-BASED DRUG DELIVERY SYSTEMS

Cell membrane-coated NPs have made a notable contribution by aiding NP-based cancer therapies in overcoming drug resistance. Due to the cell membrane structure and the retained cellular antigens, biomimetic CMCNPs extend special advantages over targeting ligand-functionalized synthetic NPs such as ligand recognition, long blood circulation, homotypic targeting, immune escape, and the ability for a sustained drug delivery. CMCNPs address the limitations of cell sensitivity, cell differentiation and cell toxicity seen in cell-based NP drug delivery by utilizing therapeutically significant cell membrane proteins instead of the cell itself. Among the various motives considered in preparing a NP-mediated delivery of therapeutic drugs, having a longer circulation time will have a major clinical impact as it increases the chances of sustained drug delivery and targets the tumor site with active and passive mechanisms such as EPR effects and evasion of reticuloendothelial system (RES) clearance^[40]^. Exploiting these mechanisms with more specific targeting and biomodulation are the major advantages provided by the biomimetic CMCNP. Listed in Table 1 are major applications of CMCNPs, and advantages reported of specific cell membrane components (ligands) over conventional targeting carrier preparations such as antibody- or peptide-decorated NPs.

There is an increasing trend in using cell membrane-coated NPs because of their advantages, including circulation time, RES, and other aspects. The first CMCNPs reported were developed by a coating of erythrocyte membrane on poly(L-lactic)-co-(glycolic acid) (PLGA) NPs, which increased NP retention in the blood to 72 h in comparison to 15.8 h of conventional synthetic stealth featured PEG-coated NPs. This was possible by avoiding the clearance by macrophage engulfment via an endogenous CD47 marker present on the red blood cell (RBC) membrane^[41]^. Also, RBC membrane-mimetic PLGA NPs with perfluorocarbon core (PFC-PLGA-RBCM) were used in membrane camouflaging to deliver oxygen to solid tumors, showing another application of cell membrane-coated NPs delivery to improve the blood circulation time by mimicking the cell surface functionality in navigating and reaching the specific target via the EPR approach due to their nano-size^[17]^.

A few other hybrid approaches such as fusing two different natural cell membranes have also been reported, e.g., RBC and platelet cell membranes fused and used for coating PLGA NPs^[43]^. This approach not only improves the circulation by inhibiting RES, but also has an added functionality of the platelet cell membrane. This membrane has cancer cell specific binding molecules such as P-selectin and CD44 receptor with surface specific capture by cancer cells, providing a significant targeting advantage over a bottom-up preparation approach to target cancer cells. Hybrid approaches of using RBC membranes and various cancer cell membranes have also been shown to inhibit tumor growth. Strategies using copper sulfide NP coating with RBCs and melanoma cancer cell membrane fused hybrid vesicles have exhibited immunosuppression mechanisms such as macrophage phagocytosis inhibition and homotypic targeting achieved by homologous surface adhesion domains on the cancer cell membranes^[52,53]^. A nanosponge made of RBC-coated PLGA NPs with α-toxin incorporation reduced staphylococcal α-hemolysin when administered to a toxin-challenged mouse showing the capability of arresting toxins by taking advantage of the biomimicking nature of RBC membranes in avoiding immune clearance^[54]^. Cell membranes increase the efficacy of cancer therapy and are also used as a cell mimicry-based delivery, which is promising in toxin neutralization.

There are other approaches being explored by CMCNPs using cancer cell membrane-coated NP-mediated cancer therapies. Capability to induce immune responses by stimulating immune cells or by blocking major checkpoints in immune pathways of cytotoxic T cell-associated proteins and other immunomodulatory molecules can be achieved using cancer cell membrane-coated NPs. Cancer cells evade elimination and survive various challenges from the immune system via loss or dysregulation of major histocompatibility complex expression (MHC) as well as decrease in immunogenicity or immunosuppression by taking advantage of anergic pathways of tumor-infiltrating lymphocytes and other active sites of “immune privilege” formed by tumor-infiltrating lymphocytes^[20]^. Along with the forementioned evasive strategies, tumor cells agglomerate congregating in a solid tumor via strong adhesion using their surface membrane proteins, which makes the infiltration restricted. In addition, unorganized and poor vasculature inhibits them from utilizing the EPR effects of NP delivery in some cancer types. Taken together, these unique features of cancer cell membrane components can be employed in developing CMCNPs with better therapeutic outcomes.

Taking into consideration of the advantages of the homing abilities and decreased immunogenic profiles of cancer cell membranes, they have the potential to enhance cancer-targeted drug delivery. To that regard, Cao *et al.*^[8]^ explored the interaction of the macrophage α4 protein and VCAM-1 of metastatic cancer cells to deliver cytotoxic anticancer drugs for metastatic inhibition in breast cancer metastasis to the lungs. In another study, Fang *et al.*^[45]^ showed that cancer cell membrane-coated NPs exert homotypic tumor targeting by means of galectin-3 and Thomsen-Friedenreich antigen (T antigen) adhesion properties of cancer cell membranes. Also, use of cracked cancer cell membrane-coated magnetic iron oxide NPs for targeting tumors showed an increased ability for homing to homologous tumors *in vivo* over other active targeting strategies, which depend on recognition selectivity and receptor density^[55]^. There was a 40-fold and 20-fold increase in uptake of cancer cell membrane-coated NPs in comparison with RBC-coated NPs and bare PLGA cores, respectively, in MDA-MB-435 cells, showing the affinity of cancer cell membrane-coated NPs towards cancer cells in attribution to the cancer cell adhesion molecules from cancer cell membranes^[55]^. In addition to drug delivery and targeting applications, the membrane coating approach has emerged as a great tool for the development of cancer vaccines. In a combinatorial therapy, Kroll *et al.*^[23]^ encapsulated an immunological adjuvant, CpG oligodeoxynucleotide 1826 (CpG) into a PLGA nanocore, which can trigger maturation of antigen-presenting cells, and the PLGA-CpG NPs coated with B16-F10 mouse melanoma cell membrane containing tumor-associated antigens improved immune responses and eventually could be developed as a vaccine towards various cancers (e.g., in the melanoma cancer vaccine approach presented in this study).

An immune cell membrane with its receptor-mediated immunogenicity and cytotoxicity can act towards a synergistic effect of immune responses and sustained chemotherapy drug release via NPs. Leukolike vectors (LLV) recently developed using neutrophil membrane-coated NPs (NM-NP) for targeting metastatic niches showed two- to threefold increased accumulation in the metastatic foci in comparison with those of bare NPs and PLGA-PEG-NPs, respectively^[11]^. This affinity towards metastatic niches is facilitated by the Mac-1, N-cadherin and other adhesive proteins expressed on neutrophil membranes available on CMCNPs over conventional PEG coating used for increasing circulation half-life and avoiding clearance^[56,57]^. In another study, cytotoxic T lymphocyte membrane-coated NPs in combination with low-dose irradiation were used for targeting and treating gastric cancer^[46]^. The low-dose irradiation increased the chemoattractant such as IFN-γ and upregulated adhesion molecule expression facilitating the increase in CD8+ T cells in the tumor environment along with homing and localization of T lymphocyte membrane-coated PLGA NPs by avoiding opsonization via highly abundant proteins such as CD45 and CD3z. It also showed a 50%−75 % decrease in phagocytic uptake of T lymphocyte membrane-coated PLGA NPs when compared to the bare NPs due to vascular extravasation by LFA-1 or CD11a. Interestingly, another study reported capabilities of T lymphocyte membrane-coated PLGA NPs to avoid being sequestered by lysosomes and to retain their lymphocyte coating, whereas plain NPs were seen to be trapped among the endolysosomal compartments prone for degradation in *in vivo* studies^[58]^. This study also found T lymphocyte-coated NPs having a twofold increase in particle density across tumors in mice in comparison to bare NPs. Many other research groups are working towards utilizing the cell membrane’s innate abilities for development of biomimetic drug carriers for cancer therapies.

## CONSIDERATIONS AND LIMITATIONS OF CELL MEMBRANE-COATED NANOPARTICLES

Cell membrane coating of NPs is employed for its various features such as targeting, immune evasion, signal transduction and other therapeutic advantages over the bottom-up formulation of targeting NPs. To take advantage of CMCNPs in cancer treatments, the cell membrane’s functional and structural features should be intact before coating drug carriers. Stability of the cell membrane is a crucial factor that determines the overall durability of the drug carrier system. Under natural conditions, cells and particles are subjected to shear and torque forces from circulation and tissue microenvironment. Cells cope with those forces by actively modulating their ligand, ligand density, lipid profile and cytoskeleton-membrane interactions. One challenge to mimicking the natural cell membrane by CMCNPs is the intracellular cytoskeleton and membrane interaction by the cells. For instance, intracellular protein-cell membrane interaction enhances the robustness of natural cells. Cell membranes are losing and/or changing some of the key cell membrane stability regulators during the membrane isolation procedures. Therefore, assessment of overall membrane stability in cell membrane-based drug delivery platforms becomes a necessity before moving forward to biomimetic-based therapy applications. Although various tools have been developed to check the membrane stability of biomimetic drug carriers, only very limited data are available on whether CMCNPs are stable enough to cross biological barriers to reach target tissues, an important point for cell membrane-based drug delivery applications^[59]^. Various techniques are reported in the literature to assess the stability of membrane structures. For instance, cryo-TEM, lipophilic dye enhanced/advanced fluorescence and/or spectrophotometric techniques are very useful for visualizing morphology and structural integrity of cell membranes^[60,61]^. RBC membranes fused with gold NPs showed stability for over 3 days in PBS and 100% serum along with an antibody binding assay showing the intact CD47 RBC cell membrane proteins on the gold-coated NPs^[13]^. In the same study, FITC-thiol conjugation to gold NPs was studied for the protection ability of membrane coating; there was no decrease in fluorescence activity over a period of 72 h, confirming the shielding effect and overall stability of cell membrane coating onto NPs^[13]^. Similarly, Tian *et al.*^[14]^ prepared stem cell membrane-coated paclitaxel-loaded PLGA NPS for cancer therapies and tested NPs for stability by monitoring change in size in FBS and drug release in physiological conditions; stable size over 72 h and low drug release compared to uncoated PLGA NPs were observed^[14]^. This stability shows the potential of cell membrane-coated NPs for formulating stable drug delivery systems. Furthermore, contemporary techniques such as microfluidic electroporation of NPs in combination with cell membrane-derived vesicles can help achieve higher stability. For such implementation, proteins from cell membranes did not get adsorbed by the NPs during the microfluidic electroporation-based coating and had improved therapeutic efficacy than did conventional extrusion-based cell membrane coating, which reveals the potential of robust techniques for biomimicry-based drug carriers^[59]^. In terms of membranes’ elastic and/or mechanic integrity, ektacytometry might be the right tool to measure membrane elongation under fluid shear stress^[62]^. The lipid composition of the cell source is also a determining factor for overall stability of CMCNPs. In one study done to compare the lipidomic profiles in cells, primary cell cultures showed higher unsaturated phospholipids in comparison with other cultured cell lines, and each cell type revealed a unique lipidomic profile, which can be employed to assess the stability of cell membranes for further improvement in membrane coating dynamics and stability of coated membranes, among which the latter is vital for mimicking cellular interaction^[60,61]^. For this purpose, colorimetric lipid assay, FTIR or X-ray scattering are useful tools for the qualitative phospholipid assessment^[63,64]^.

It is important to consider the cellular source and state to avail the cell membrane characteristics. In this manner, Evangelopoulos *et al.*^[65]^ showed the cell source to be a determinant factor for the immunogenicity of biomimetic NPs. In their study, multistage cell membrane derived vesicles from different sources were analyzed in terms of opsonization and phagocytosis as well as targeting of inflamed tissues. Results revealed that cellular coating derived from a syngeneic cell membrane source resulted in higher avoidance of uptake by the liver and immune repertoire cells^[65]^. On the other hand, healthy homotypic cells in their growth phase with intrinsic properties of their membrane should be preferred for cell membrane isolation and coating of NPs. Homogeneity of the clonal population of cells is vital to interpret the true therapeutic efficacy of cell membrane coated NPs. To meet this demand, expression levels or quantification of certain surface markers (ligands or receptors) becomes a pivot point. Cellular state and homogeneity of cell membranes can be assessed via solid techniques such as SDS-PAGE, Western Blot, and flow cytometry, which might be very useful to serve the abovementioned purposes^[66]^. This evaluation step of characterizing the cells for their biomarkers and other ligands that will be used in targeting, signal transduction and other therapeutic approaches will improve translational effects.

As CMCNPs confer their therapeutic properties via membrane protein interactions with the local microenvironment and other cells, the prominent presence of the desired cell membrane proteins needs to be maintained in the cell culture. Various chemical signaling and transfection strategies can be employed to induce desired protein expression and regulation of cellular states in cultured conditions. On the contrary, the long-term cell culture of some specific cell types might adversely affect their desired nature for CMCNP applications. For instance, the cell culture condition determines the mesenchymal stem cells’ (MSCs) phenotypes, and this makes them heterogeneous between individuals, cell populations and even batches^[67]^. *In vitro* expansion of MCSs affects their mRNA expression profiles, and surface proteins responsible for migration and/or adhesion (i.e., CXCR4, CXCR7, C-met/HGF, *etc*.) are also affected^[68–71]^. In the case of immune cell membrane coating of NPs, cellular source and state of immune cells are important as they undergo various changes with respect to pro- and postinflammatory phases, e.g., M1 and M2 macrophages which represent pro- and post-inflammatory conditions with plausible related membrane proteins. Tumor-associated macrophages (TAMs), for example, are very similar to M2-macrophages phenotypically^[72]^, and support cancer cells in the tumor microenvironment^[72,73]^. Membranes derived from these cells might help tumor growth and shadow effects of the chemotherapeutic cargo. Therefore, following the cell source, state and membrane characterization, characterization of membrane-coated NPs via aforementioned solid and fundamental techniques becomes a touchstone and can reveal the cell membrane characteristics on NPs^[62]^.

For these reasons, it is getting more attractive to engineer the surface of well-defined cells with desired molecules (peptides, proteins, or small molecules) via genetic or non-genetic techniques before harvesting cell membranes^[74]^. In this case, the natural receptor profiles of cell membranes are getting affected, and the results of this effect are not fully known so far. For example, highly biotinylated erythrocyte membranes are prone to bound C3b proteins and getting phagocytized by macrophages via complement activation. It is speculated that biotinylation inactivates the “self-markers (i.e., CD47)” or complement regulators (i.e., CD59) on the cell membrane^[75–77]^. On the other hand, researchers have reported that NP conjugation on the cell membranes does not fully affect their natural behaviors. Stephan *et al.*^[59]^ conducted a comprehensive analysis of NP-tethered T cells in terms of cell division, antigen pulse, transmigration, and synapse formation. Maleimide-thiol conjugation of NPs on the cell membrane until a certain number of NPs (~100 NPs/cell) did not interfere with their physiological tasks^[59]^. Overall, immunogenicity of the engineered cell membranes is highly dependent on the cell source selection, methods used for engineering, and the modification degree of the membranes. Therefore, comprehensive qualification and characterization of CMCNPs via the abovementioned fundamental and engineering methods are crucial for the translation of biomimetic-based drug delivery applications to the clinic.

Although cell membrane-coated nanocarriers have great prospects to deliver cytotoxic drugs at the desired location for tumors, there are several limitations and challenges associated with this strategy. The cell membrane is comprised of various proteins, some of which are required for targeting specificity and evading immune responses while other abundant proteins have other interactions in the host environment whose biodistribution, immune responses and toxicity profiles have not yet been elucidated. Cell lines required for extracting cell membranes need a high quality control to avoid variation and to maintain their homogeneity; for instance, stem cells with heterogeneous populations have been observed in a clonal cell population^[78]^. Cell membrane isolation procedures, however, are not robust and are often limited to laboratory settings, which can be a challenge in clinical translation of the CMCNPs. In addition, OMVs extracted from bacteria involve a tedious process, and any external stimuli including iron depletion, oxidative stress, temperature stress and genetic manipulations during the process can give rise to undesirable changes in OMV composition^[79]^. Therefore, quality control of protocols that could retain functional and structural aspects of cell membranes or OMV during membrane isolation techniques needs to be established. In the case of a whole-cell delivery system, it is still challenging for carrier cells to have site-specific drug releases based on many factors including their survival, drug retention of NPs loaded in cells, and other pharmacodynamic effects from the drug release of NPs on their journey to reach the target. The potential side effects of these endogenous carriers are still under investigation. On the other hand, some of the functionalized NPs have already entered clinical trials, and the cell membrane-coated NPs have shown promising results in preclinical studies with a potential for testing in clinical trials, indicating the urgency for the development of regulatory and safety guidelines for a smooth clinical translation^[80]^.

## CELL TYPES USED IN CELL- AND CELL MEMBRANE-BASED DRUG DELIVERY APPLICATIONS FOR CANCER THERAPY AND IMMUNOMODULATION

It is evident from the recent CMCNP applications that cell membrane coating is highly investigated for biomimetic approaches in drug delivery^[81–83]^. Unique drug delivery features such as the ability to reach solid tumors and homing to inflamed tissues can be achieved through CMCNPs via coating of NPs with membranes of different cell types including RBCs, platelets, lymphocytes, cancer cells, and others. Among those considered, having a prolonged blood circulation time, a specific type of tumor accumulation through adhesion molecules, and other specific tumor microenvironment interactions are highly desired. One of the major advantages of using CMCNPs is also to overcome drug resistance incurred by tumor heterogeneity. A tumor-homing cell membrane employed in CMCNPs can help NPs navigate across the interstitial fluid pressure, avoid TAM uptake, target different cancer populations, and achieve higher drug concentrations in those targeted cells, thereby circumventing drug resistance seen with low drug bioavailability in conventional chemotherapies^[84]^. The type of cell or cell membrane choice is critical to take advantage of site-specific delivery and targeting via camouflaging, and reduction in unfavorable interactions with complementary systems *in vivo*. Cells are used in drug delivery through various approaches including NP-mediated autocrine signaling for manipulating cellular responses at targeted sites, hitchhiking of NPs on cells, cell surface engineering for immunomodulatory responses, and cell membrane coating of NPs for multifunctional therapeutics [Figure 3]. Major cell types used for CMCNP-mediated cancer therapies and potential immunomodulatory effects are listed in Table 2.

### T cells

T cells have a variety of immune functions within the body because of the large amounts of proteins found on their membranes. T cell proteins have been found to affect MPS uptake, to be involved with immune tolerance, and to target endothelium/tumors^[85]^. The combination of T cell targeting and permeability abilities along with NP modulation makes T cells attractive candidates for cancer therapies. Stephan *et al.*^[100]^ have reported synapse directed delivery of immunomodulator drug NSC-87877 via T cell surface-conjugated NPs. In this research, NPs attached to T cell membrane proteins facilitated molecular interactions regulating the prevention of autoimmunity while boosting immune responses against tumor cells. In this study, lipid NPs prepared from maleimide-functionalized liposomes through extrusion and conjugated to effector T-cells via incubation were injected into prostate cancer implanted mice. The liposome-treated T cells managed a 5.2-fold reduction in tumor volume and gave a survival advantage of over 14 days over the mice treated with just T cells. As an alternative approach to NP hitchhiking via T cell surface or compartment, our group showed that T cell membrane-coated NPs actively target tumor regions via their specific T cell receptors called TCR^[15]^. In this study, chemotherapeutic drug Trametinib loaded PLGA NPs were coated with the membranes of melanoma-specific “anti-gp100/HLA-A2” T-cell receptor (TCR) bearing T cells. T cell membrane-coated NPs (T-MNPs) showed high stability and cyto- and hemocompatibility. T-MNPs produced a threefold increase in cellular uptake towards the melanoma cell line *in vitro* compared to bare PLGA NPs. The *in vitro* cancer killing effectiveness of T-MNPs was significantly higher compared to other NP groups. An animal study was followed by administering T-MNPs to DM-6 tumor-bearing mice. *In vivo* biodistribution studies demonstrated the targeting capabilities of the T-MNPs with more than a twofold increase in tumor retention compared to the uncoated and nonspecific membrane-coated groups. Mitchell *et al.*^[12]^ showed that TRAIL-coated lymphocytes killed cancer cells in circulation. Cancer cells and blood cells use a selectin-based adhesion to interact between each other. With this knowledge, liposomes with E-selectin adhesion proteins and TNF-related apoptosis-inducing ligand (TRAIL) were presented on its surface and conjugated with lymphocytes to provide T cells with the ability to adhere and improve cytotoxic effects in circulating colon or prostate tumor cells in the bloodstream^[12]^. Control mice had ~130,000 cancer cells/mL of blood while ES/TRAIL-coated lymphocyte treated mice had < 2,000 cancer cells/mL. Further experimentation revealed a decreased number and increased apoptosis of COLO 205 cells lodged in mouse lungs after ES/TRAIL liposome treatment. The lymphocytes were still functional even after 2.5 h of circulating in the mice displaying the feasibility of these NPs for cancer therapy^[12]^.

In addition to T cells, there are other types of lymphocytes that show potential in treating tumors by providing effective targeting. For instance, Parodi *et al.*^[58]^ used lymphocyte properties to investigate the particle uptake between host and donor cells and the accumulation of LLVs in melanoma tumors due to the intrinsic property of lymphocytes, which can cross biological barriers and accumulate at target tissues. They also showed that nanoporous silicon particles can perform similar actions when coated with cell membranes from human THP-1 and murine J774 cells. A decrease in uptake for the LLV coating was observed when the donor membrane matched the host membrane ranging from ~50% to ~75%. Murine J774 LLVs had delayed accumulation in the liver for up to 40 minutes *in vivo* and also bound to the outside of murine B16 melanoma tumors in a non-destructive manner^[58]^. These studies show the potential of T cells in CMCNPs for their targeting, cytotoxicity, and immunogenicity towards tumors.

### Macrophages and dendritic cells

Macrophages are a type of immune cell that secrete cytokines and chemokines to home in monocytes to deal with damaged tissues and/or infections. Macrophages are known to cross biological barriers and are present in tumor masses, making them potential drug delivery carriers. In tumor sites, macrophages can release chemo-attractants to attract more macrophages, enhancing immune responses. Furthermore, macrophages can operate under hypoxic conditions, which is commonly seen in tumors. Xie *et al.*^[101]^ developed a drug delivery and theragnostic system using macrophages as NP carriers. Biodegradable photoluminescent poly (lactic acid) (BPLP-PLA) were loaded with anti-BRAF V600E mutant melanoma specific drug (PLX4032) and conjugated with muramyl tripeptide (MTP) (MTP-BPLP-PLA-PLX4032), which were internalized in THP-1 macrophage cells. THP-1 cells and MTP-BPLP-PLA-PLX4032 NP-loaded THP-1 cells were co-cultured with 1205Lu cells and WM35 cells as models for high and low metastatic melanoma cells, respectively. Confocal microscopy and quantitative flow cytometry analysis revealed that the NP-loaded THP-1 were able to bind to 1205Lu cells under static conditions and release the NPs via exocytosis after binding to the cancer cells. Under dynamic shear-flow conditions, NP-loaded THP-1 cells bound to WM35 cells and over 90% of 1205Lu cells as confirmed by flow cytometry. Another *in vitro* study was conducted to show the ability for the drug delivery system to effectively kill melanoma cells. A minimum of 5,000 cells of NP-loaded THP-1 cells significantly decreased the viability of 1205Lu and WM35 cells compared to normal THP-1 cells.

Combined with drug-loaded NPs, macrophages and their membranes can serve as a potent drug delivery vehicle. For instance, Xuan *et al.*^[87]^ prepared doxorubicin (DOX)-loaded mesoporous silica nanocapsules (MSNCs) camouflaged with macrophage cell membrane (MPCM) for treatment of tumors. More than 30% of MPCM-camouflaged MSNCs were phagocytized by macrophages, and MPCM-camouflaged MSNCs remained in circulation after 24 h and 48 h of treatment with 36% and 32% retention, respectively, whereas all bare MSNCs were cleared after 24 h. An *in vitro* study showed that 4T1 mouse breast cancer cells efficiently take up DOX-loaded MPCM-camouflaged MSNCs. An animal study was followed by injecting DOX-loaded MPCM-camouflaged MSNCs and regular MSNCs into 4T1 tumor-bearing mice. After 72 h, MSNCs aggregated largely in the spleen and liver, whereas some accumulation of MPCM-camouflaged MSNCs were at the same sites. However, DOX fluorescence could be detected in the tumor site because of the accumulation of MPCM-camouflaged MSNCs at the tumor site. Tumors treated with DOX-loaded MPCM-camouflaged MSNCs had their volumes decreased and, in some cases, regressed almost completely^[87]^.

Similarly, Thamphiwatana *et al.*^[88]^ prepared macrophage mimicking NPs (MM-NPs) by using J774 mouse macrophage cell membranes and fusing them with the PLGA core for treatment of *E. coli* infections. The MM-NPs contained LPS-binding proteins such as CD14 and TLR4 along with cytokine-binding receptors CD126, CD130, CD120a, and CD120b. The LPS removal capacity of the MM-NPs was measured using assays, which indicated 62.5 ng of LPS were removed per milligram of MM-NPs. The MM-NPs were then measured for their ability to sequester proinflammatory cytokines in a mixture, where they removed 105.1 pg of IL-6, 4.3 pg of TNF, and 6.5 pg of IFN-γ, corresponding to 52.5%, 11.6%, and 14.8%, respectively. Further study revealed 60% of mice survived lethal LPS levels. A lethal dose of *E. coli* was administered to mice followed by MM-NPs treatment, and 4 out of 10 mice survived up to 60 h. The number of bacteria in key organs and proinflammatory cytokines were significantly lower compared to the control treatment^[88]^.

Dendritic cells (DC), on the other hand, have the ability to express ligands capable of activating various immune cells such as natural killer (NK) cells responsible for killing tumor cells. DC are often the target for antigen-presenting vaccines, but these cells can also be repurposed for immunomodulation. Dendritic cells can process a variety of antigens, and as shown by Munich *et al.*^[86]^, exosomes derived from TNF, FasL, or TRAIL expressing DC directly kill tumor cells and activate NK cells via TNF superfamily ligands (TNFSFLs). TNFSFL-expressing mature DC exosomes (mDCex) and TNFSFL-expressing immature DCex (iDCex) expressed approximately 300 pg of TNF and < 1 pg of FasL per 100 μg of intact iDCex, and 800 pg of TNF and 2 pg of FasL per 100 μg of intact mDCex on their respective membrane surfaces. mDCex exposed to melanoma cells through incubation induced significant cell death at 24 h and further increased at 48 h and 72 h. mDCex were also capable of killing other cancer cell types such as lung carcinoma KLN205 cells and colon carcinoma MC38 cells. NK cell exposure to mDCex and iDCex showed greater NK cell activation when exposed to mDCex than iDCex^[86]^. In another study, Krishnamurthy *et al.*^[89]^ synthesized nanoghosts by using a monocyte cell membrane shell and DOX-loaded PLGA core. Using flow cytometry, the uptake of the DOX-loaded nanoghosts by MCF-7 breast cancer cells was higher than that of PLGA NPs. The cytotoxicity of the nanoghosts was evaluated on MCF-7 cells. Blank nanoghosts and plain PLGA NPs showed no cytotoxic effect after a 72-h treatment period. An MTS assay was then conducted to measure the killing effectiveness between free DOX, DOX-loaded nanoghosts, and DOX-loaded PLGA NPs. The IC_50_ values for DOX-nanoghosts and DOX-PLGA were 4 μmol/L and 12 μmol/L, respectively. Macrophages, DC, and monocytes together considered as the mononuclear phagocyte system (MPC) have overlapping characteristics and robust immune responses towards the tumor niche, which can be employed in the development of CMCNPs for cancer therapies^[102]^.

### Neutrophils

Neutrophils like their other white blood cell counterparts, macrophages and T-cells, play an active role in the body’s immune system and have similar advantages for therapeutic purposes. Neutrophils set themselves apart from the other cell types for having the capability to travel to places in the body that other cells cannot access, such as the brain^[103]^. For example, neutrophils have the capability to penetrate inflamed brain tumors as reported by Xue *et al.*^[90]^, who used neutrophil-mediated anticancer drug delivery for suppression of malignant glioma. In this research, neutrophils carrying paclitaxel (PTX)-loaded liposomes suppressed glioma in mice. The inflammation signals facilitated neutrophils to release PTX cationic liposomes (PTX-CL) into the tumor cell. PTX-CL and neutrophils were incubated together so the liposomes could be taken up by the neutrophils. The PTX-CL/neutrophils (PTX-CL/NEs) had a loading capacity of 18 μg PTX/10^6^ cells. PTX-CL/NE showed delayed accumulation in the liver compared to Taxol and PTX-CL. There was high accumulation of PTX in the spleen due to the natural migratory patterns of neutrophils. PTX-CL/NE brain-targeting efficiency was determined to be greater than one showing the efficient targeting abilities of PTX-CL/NEs. PTX-CL/NE treatment ensured a 50% survival rate of up to 61 days in treated mice compared to Taxol and PTX-CL, which showed 29- and 38-days survival, respectfully. About 25% of mice treated with PTX-CL/NEs survived more than 4 months^[90]^. In another study, Kang *et al.*^[11]^ developed a nanosized neutrophil-mimicking drug delivery system (NM-NP) by coating neutrophil membranes onto carfilzomib-loaded PLGA NPs to neutralize circulating tumor cells (CTCs) and to inhibit formation of the metastatic niche when NM-NPs loaded with carfilzomib (NM-NP-CFZ) and CTCs were incubated together. *In vivo* studies of mice implanted with 4T1 lung cancer cells followed by NM-NPs, PLGA-PEG-NPs and bare NPs showed that NM-NP accumulation in the metastatic foci increased 2.12- and 3.02-fold compared to bare NPs and PLGA-PEG-NPs, respectively, after 24 h of intravenous administration. NM-NPs showed a strong attraction to the liver and spleen. NM-NP-CFZ were administered to mice four times on 0, 7, 14, and 21 days. Early metastatic nodule formation was found to be significantly low. The same group showed that NM-NP-CFZ reduced 4T1 metastasis foci by 87.2% in animal studies^[11]^.

### Red blood cells

Red blood cells (RBCs) are the major cells in circulation providing longer circulation and eventually higher accumulation as well as targeting of tumors^[104]^. Furthermore, RBCs can be type-matched to enhance biocompatibility^[104]^. Their availability and lack of intracellular organelles makes their membranes easy to be collected for coating onto drug-loaded NPs. RBC membranes with longer circulation characteristics combined with drug-releasing NPs can lead to prolonged drug release. Luk *et al.*^[7]^ combined RBC membranes and DOX-loaded PLGA NPs [RBC-NP(DOX)] as an anti-tumor drug delivery system. RBC-NP(DOX) killing effectiveness was tested on EL4 mouse lymphoma cells with an *in vitro* cytotoxicity assay with a 72-h incubation period. While cancer cell killing tests revealed free DOX working more effectively with an IC_50_ of 1.4 ng/mL compared to the RBC-NP(DOX) with an IC_50_ of 5.6 ng/mL, the latter formulation had better uptake by EL4 cells compared to free DOX. Mice were implanted with lymphoma T cells (EL4 cells), which were allowed to grow for 9 days and then treated with RBC-NP(DOX). The formulation-controlled tumor growth almost doubled the median survival from 24 days to 47 days^[7]^.

Besides cancer therapy, RBC membrane coating has also been used in vaccines and toxins. Toxoid vaccines are developed from the inactivated toxins, but the processes used for preparation of these vaccines are difficult and denaturing for the toxin protein structure, leading to altered antigen presentation, and compromising immunogenicity. NP-based toxoids (nanotoxoids) can circumvent these preparation challenges by detaining the toxins inside the cell membrane and delivering them safely *in vivo.* Wang *et al.*^[91]^ developed a NP-based anti-virulence vaccine to target methicillin-resistant staphylococcus aureus (MRSA) skin infections. RBC membranes were fused to the surface of PLGA where the RBC membrane coating was the functional part for pore-forming α-hemolysin (Hla) (heptameric cell membrane pore-forming factor) insertion. In this study, nanotoxoid (Hla) injections in mice showed induction of Hla-corresponding antibodies and germinal center formation characteristic in draining lymph nodes. There was almost no drop in anti-Hla titers over a five-month period. When nanotoxoid (Hla)-vaccinated mice were challenged with MRSA bacteria, there was clear attenuation of lesion formation with a 5-fold decrease in dermonecrotic area and inhibition of Hla-mediated skin damage, showing high extravascular neutralization activity of the titers produced from the vaccine induction^[91]^. Similarly, Hu *et al.*^[54]^ developed another biomimetic approach where RBC-based nanosponges were shown to absorb pore-forming toxins (PFTs), which are the most common toxic proteins generated by pathogenic bacterial infection.

RBCs also contain characteristic membrane proteins that protect them from macrophages. Coating gold NPs (AuNPs) with RBC membranes via extrusion showed effective shielding of AuNPs from phagocytic uptake as shown by Gao *et al.*^[13]^ who incubated RBC-AuNPs with J774 murine macrophage cells. Cell uptake of RBC-AuNPs was measured after 30 minutes. RBC-AuNPs had an uptake of 3.2 ng/1,000 macrophage cells. Uncoated AuNPs had an uptake of 13.5 ng/1,000 macrophage cells. RBCs coexist with immune cells in circulation where they might possess abilities in maintaining homeostasis in the circulating cells. In that regard, Danesh *et al.*^[105]^ reported the use of RBC exosomes which triggered monocytes to release proinflammatory cytokines for boosting lymphocyte responses *in vitro*. Mixing these cells with extracellular vesicles (EVs) results in the secretion of proinflammatory cytokines and increased survival of peripheral blood mononuclear cells (PBMCs). EVs also increased CD4^+^ and CD8^+^ T cell proliferation. PBMCs from 0, 21, and 42 days were cultured with EVs for 24 h, and on day 0, EVs induced significant upregulation of various cytokines, especially IL-1β, as compared to those incubated for 21 days and 42 days. EVs incubated with both monocytes and T cells interacted with monocytes instead of T cells, and this interaction induced the production of TNF-α via exosomes^[105]^. Overall, RBCs possess intrinsic properties of longer circulation times, phagocytosis avoidance, and stimulation of immune cells, which may be more advantageous, while developing cancer and immunomodulation therapies.

### Platelets

Platelets are small circulating cells with a lifespan of around 8–10 days. Their primary role is to maintain homeostasis in vascular injury by sealing ruptured vessels and releasing granules that promote angiogenesis and recruitment of regenerative cells^[106]^. Platelets also play a role in tumor growth and metastasis through interacting with tumor cells and the tumor microenvironment where they can adhere to cancer cells through GPIb-IX-V, GPIIb-IIIa, P-selectin, and tumor cell integrin αvβ3^[107]^. Researchers have incorporated proteins on platelets and used these engineered platelet cell membranes for coating NPs. For instance, Wang *et al.*^[92]^ conjugated anti-programmed-death ligand 1 (aPD-L1) on the surface of platelets to reduce post-surgical tumor recurrence and metastasis. Upon activation of the platelets, platelet microparticles are generated with aPD-L1-conjugated membranes. *In vivo* studies were conducted on mice bearing primary melanomas (B16-F10) or triple-negative breast carcinomas (4T1). The study revealed that B16F10-inoculated mice treated with platelet-PD-L1 had the smallest relapsed-tumor volumes compared to free aPD-L1, platelets, and PBS. In their B16F10 metastatic study, platelet-PD-L1 reduced both local tumor recurrence and lung metastasis, whereas free PD-L1 only reduced metastatic cancer. Another metastatic study was done with 4T1 carcinomas, where the platelet-aPD-L1 group showed few metastatic foci compared to 16 foci for the free aPD-L1 group and ~30 foci for the platelet and PBS group^[92]^. Taken together, these results show the potential of platelet cell membrane engineering in developing CMCNPs.

Due to a lack of a nucleus, platelets cannot be directly engineered. Instead, researchers engineer megakaryocytes, which then release engineered platelets. Li *et al.*^[93]^ utilized that strategy to engineer platelets to express membrane-bound TRAIL to induce apoptosis in tumor cells. The TRAIL-expressing platelet-like particles (TRAIL-PLPs) exhibited cytotoxicity against the cancer cells, reducing cell viability to approximately 20% when using a TRAIL-PLP concentration of 10 μg/mL. A TRAIL-PLP concentration of 1 μg/mL reduced cell viability to 50% and 30% for MDA-MB-231 and PC3 cell lines, respectively. *In vivo* studies revealed that TRAIL-expressing platelets reduced metastases in the liver compared to empty vector transduced platelets^[93]^.

Researchers have also used platelet membranes coupled to NPs to enhance NP circulation time because of the abundance of platelets found in the circulation. Hu *et al.*^[94]^ developed a platelet membrane-coated NP platform (PM-NP) to target and inhibit myeloma cells. These PM-NPs were designed to deliver bortezomib (bort) and use alendronate (Ald) as the targeting ligand. For the *in vitro* study, they treated myeloma cell line NCI-H929 with PM-NP-bort and Ald-PM-NP-bort. PM-NP-bort and Ald-PM-NP-bort exhibited late apoptosis rates of 38.6% and 37.4%, respectively^[94]^. Also, PM-NP-bort and Ald-PM-NP-bort had IC _50_ values of 13.6 ng/mL and 13.1 ng/mL respectively, but was lower than NP-bort IC_50_ of 23.2 ng/mL^[94]^. Li *et al.*^[108]^ designed silica NPs coated with TRAIL-conjugated platelet membranes for the treatment of CTCs *in vitro.* The authors showed that TRAIL-PDMV-Si particles reduced MDA-MB-231 and PC3 cell viability to approximately 5% when the TRAIL concentration was 1 μg/mL. The *in vivo* study indicated ~40-fold reduction of lung metastases compared to TBS and PMDV-Si particle control groups. Furthermore, TRAIL-PDMV-Si particles reduced lung metastases by ~8 fold compared to soluble TRAIL^[93]^. The strengths of platelets as a drug delivery platform are their long circulation times, which allow for better targeting of cancers found in the circulation, their release of granules that can enhance immune responses, and ease of availability in the body.

### Stem cells

MSCs are used as drug delivery systems for their innate targeting ability towards inflammation and tumor-tropic properties. MSCs can penetrate solid tumors and interact with target cells. Additionally, MSCs can be genetically modified to express therapeutic genes and their expression could be enhanced with NPs. Their membranes retain most of their functionality and therefore can be employed for membrane-based drug delivery systems^[109]^. For instance, Sadhukha *et al.*^[95]^ engineered mesenchymal stem cells as tumor-targeted therapeutic carriers where they treated human MSCs with paclitaxel-loaded polymeric NPs. Nano-engineered MSCs were cytotoxic towards A549 lung adenocarcinoma cells and MA148 ovarian cancer cells *in vitro.* This was determined via MTS analysis that showed an IC_50_ of 6.71 nmol/L and 4.52 nmol/L in A549 cells and MA148 cells, respectively. Animals studies using infrared fluorescence revealed the nano-engineered MSCs initially travelled to the lung tumors but later distributed to the liver and spleen^[95]^. In addition, Gao *et al.*^[10]^ developed bone marrow derived mesenchymal stem cell membrane-coated gelatin nanogels (SCMGs) as a tumor-targeting drug delivery system. These gelatin nanogels were loaded with the anticancer drug DOX. SCMGs-DOX were incubated with HeLa human cancer cells for 24 h. An MTT assay showed an IC_50_ of 0.63 µg mL^−1^ and 2.55 µg mL^−1^ for SCMGs-DOX and gelatin-DOX, respectively. HeLa cells uptake of SCMGs-DOX was almost 100% after 0.5 and 1 h. SCMGs-DOX injected into mice bearing HeLa tumor showed delayed tumor growth for 15 days. The average tumor weight for the SCMGs-DOX treated mice was smaller compared to the four control groups: PBS, gelatin, free-DOX, and gelatin-DOX^[10]^.

MSC membrane-coated NPs also show improved targeting in brain tumors and survival in mice with no cognitive side-effects showing the capabilities of MSC coated NPs for delivering drugs across the Blood Brain Barrier (BBB). For example, Wang *et al.*^[96]^ used bone marrow-derived MSCs loaded with paclitaxel (PTX)-encapsulated PLGA NPs for glioblastoma therapy in rats. The MSC NPs were injected into contralateral brain hemispheres. MSC NPs (1 pg drug/cell) decreased C6 glioma cell survival by 40%−50% compared to 100% C6 cell survival demonstrated by untreated MCS. Two days after injecting the right brain hemisphere with Cm-Dil-stained MSC NPs, about 44.4% ± 5.4% of drug-loaded MSCs migrated towards gliomas with no abnormal consciousness or motor responses. In addition, the median survival time for the tumor-bearing mice was 35.5, 24.5, 22.0, 13.5, and 14.5 days for MSC NPs, MSC Ptx, Ptx-PLGA NPs, MSCs, and saline, respectively, with the MSC-NP group exhibiting the most significant reduction in glioma areas^[96]^. Stem cells with their ability to differentiate and produce a vast array of bioactive molecules including cytokines, chemokines, and other growth factors may impart immunomodulatory responses in tumor niches imparting therapeutic effects. Autocrine signaling and paracrine signaling pathways in stem cells towards achieving therapeutic effects on inhibiting tumors can be accomplished by tagging stem cells with signaling molecule-loaded NPs^[110]^. Also, stem cell membranes have efficient abilities to home to inflammation sites and tumor lesions, making them ideal candidates for targeting approaches in the development of CMCNPs.

### Cancer cells

Cancer cells as therapeutic carriers are unique candidates for CMCNPs as they can be easily cultured to harvest higher yields of cell membranes. The cancer cells target other cancer cells because of their high affinity within tumor interactions and they can escape the immune system for longer circulation in the bloodstream. Cancer cell membrane-coated NPs (CCMNPs) can deliver tumor-associated antigens to antigen-presenting cells and can be used in immunomodulatory, anticancer drug, or vaccine delivery platforms^[23,45,55,97]^. MDA-MB-435 cell-based CCMNPs are an example of CCMNPs for cancer therapy, which were prepared using human cancer cell line membranes with homotypic aggregation properties and coated onto PLGA with fluorescent dye loaded in the core to assess their therapeutic properties in cancer^[55]^. Results showed affinity of CCMNPs towards cancer cells in attribution to their cell adhesion molecules, which aid in homotypic binding. Sun *et al.*^[97]^ developed a drug delivery system using DOX-loaded gold nanocages (AuNs) as an inner core and 4T1 cancer cell membrane (CMVs) coating as the outer core. This type of drug delivery system (CDAuNs) utilizes the homotypic targeting of the cancer cell membranes and the hyperthermia-responsive ability of the AuNPs for thermal-triggered drug release. The DOX loading capacity and encapsulation efficiency were 5.5% ± 0.2% and 97.3% ± 0.4%, respectively. In *in vitro* studies, CDAuNs released DOX under hyperthermia and targeted 4T1 cancer cells via cell membrane interactions. An *in vivo* biodistribution study revealed that CDAuNs with or without NIR irradiation had threefold lower DOX accumulation in heart tissue compared to free DOX. Furthermore, CDAuNs reduced tumor volumes and metastatic nodules by 98.9% and 98.5%, respectively, using a 4T1 breast tumor model^[97]^. CDAuNs show the ability to combine both biological and synthetic materials for multifunctional cancer therapeutics.

Towards developing cancer vaccines, cancer cell membrane-based antigen presentation can yield a prominent immune response against tumors. Taking advantage of the antigen-presenting ability of cancer cell membranes, Kroll *et al.*^[23]^ developed an anticancer vaccine by coating CpG ODN-loaded PLGA with B16-F10 mouse melanoma cell membranes (CpG-CCNPs)^[23]^. CpG-CCNPs were successful in stimulating bone marrow-derived DC to secret interleukin (IL)-6 and IL-12 more than free CpG ODN, and CpG-CCNPs managed to induce dendritic cell maturation after *in vivo* administration. A high T-cell proliferation was observed in mice on CpG-CCNP treatment, with infiltrating T cells generating multiple tumor antigen specificities such as enhanced production of IFN-γ and IL-2. The vaccine prevented tumor occurrence for 86% of mice after 150 days of administration^[23]^. In another study, Fang *et al.*^[45]^ reported that cancer cell membrane-coated NPs (CCMNPs) can be used as an anticancer vaccine and a drug carrier. MPLA modified mouse melanoma cancer cell membranes were extruded with drug loaded PLGA NPs. These CCMNPs have a dual functionality of both tumor-antigen presentation for immunotherapy and homotypic targeting of cancer cells to deliver the drug payload. Incubation of MPLA incorporated CCMNPs with DC was shown to upregulate maturation markers such as CD40, CD80, and CD86 in the DC. Co-culture of MPLA-CCMNPs pulsed DC and splenocytes with gp100 epitope showed T-lymphocyte crowding around DC, whose activation was later quantified by IFN-γ, confirming antigen-specific response elicited by MPLA-CCMNPs^[45]^. Cancer cell membranes have various antigen components, which can be efficiently presented to DC via CCMNPs to elicit desired immune responses, and adhesion molecules on cancer cell membranes can be highly advantageous in homotypic targeting of drug-loaded NPs to cancer tissues.

### Bacteria

Bacterial outer membrane vesicles (OMVs) have been used as vaccine platforms for decades due to their ability to carry surface antigens, to be readily phagocytosed by cells, and to stimulate innate immunity and promote adaptive immune responses. In addition, bacterial OMVs have recently been investigated as therapeutic delivery systems just as OMVs and/or in combination with NPs. Bacterial OMVs are attractive for their high uniform yield. Compared to other mammalian cells and their membranes, OMVs carrying drug-loaded NPs have two potential positive therapeutic effects; immunostimulant and payload delivery. Bacterial membranes facilitate immunogenic antigens and pathogen associated-molecular patterns (PAMPs), which help to stimulate immune responses and associated pathways^[111–113]^. Gao *et al*.^[114]^ have used an *Escherichia coli (E. coli)* bacteria pathogen model to study bacterial membrane-coated NPs. Here, they collected bacterial membranes and fused them to the surface of AuNPs (gold nanoparticles) by chemical interactions. Injection of these bacterial membrane-coated AuNPs showed boosted activation of CD11c+ DC in lymph nodes with upregulation of costimulatory molecules (CD40, CD80, and CD86) as well as elicited B cell responses (increased IgG levels) and T cell responses (increased levels of IFN-γ and IL-12 on vaccination with BM-AuNPs)^[114]^. Similarly, *Salmonella enterica* OMV-expressing pneumococcal PspA were employed to probe antibody responses, and mice immunized with PspA engineered OMVs triggered immune responses in contrast with no responses coming from only OMVs or only PspA groups^[115]^.

Bacterial outer membranes have also been shown to suppress tumors such as murine colon adenocarcinoma tumors, which may explain the immunostimulatory features of bacterial membranes. In a related study, mice were transplanted with the cancer cells and treated with a genetically engineered *E. coli* outer membrane, with inactivated lipopolysaccharide (LPS)^[98]^. Naturally, LPS binds to TLR4 receptors, which in turn produces the cytokine IL-8, but the impaired LPS does not interact with TLR4 receptors^[98]^. The modified *E. coli* OMVs showed decent prevention of tumor growth for murine carcinoma and B16BL6 melanoma cells in comparison to functionalized NPs prepared using a bottom-up approach^[98]^. In similar application of OMVs by Gujrati *et al.*^[51]^*, E. coli* was engineered to express human anti-Her-2 protein with reduced endotoxicity toward human cells and with an ability to kill cancer cells by delivering small interfering RNA (siRNA) via targeting kinesin spindle protein (KSP). These OMVs had an affinity towards HER-2-overexpressing tumors, which resulted in greater tumor inhibition by 66% compared to the control group mice. Modified OMVs did not show induction of a severe immune response or prolonged inflammatory responses and were safe at higher doses^[51]^. Fantappiè *et al.*^[99]^ engineered *E. coli* OMVs to carry heterologous antigens: SpyCEP, streptolysin O, Spy0269, SAM_1372, and R-TEM b-lac. These antigens were chosen for their ability to induce immune responses, measurable functional activities, and belonging to different compartments of the cell. The recombined OMVs were capable of inducing antibody responses especially from those immunized with Slo-OMVs and SpyCEP-OMVs with > 80% survival rates in mice^[99]^. Bacterial cell membranes possess various characteristics including easy production and manipulation via molecular biology techniques, affinity to hypoxic areas and other intrinsic tumor suppressing abilities, which can aid in the development of safer vaccines and exhibit immunomodulatory effects *in vivo* when combined with conventional NP drug delivery systems.

## CELLULAR COMPONENTS ASSOCIATED WITH CELL- AND CELL MEMBRANE-BASED PAYLOAD DELIVERY

### Cell membrane components for active payload targeting and delivery

#### CD47

CD47 (cluster of differentiation 47) is also known as an integrin-associated protein, which is ubiquitously expressed in the transmembrane of human cells and is part of the immunoglobulin family with 60%−70% similarity among mice, rats, and bovine CD47. CD47 has a molecular weight of 50 kDa and is composed of a 109-amino acid long membrane receptor with an extracellular N-terminal IgV domain, five transmembrane domains and a short C-terminal intracellular tail, and it interacts in *cis* and *trans* with integrins and signal-regulatory protein alpha (SIRPα)^[116]^. The immune system recognizes invaders as foreign because they express determinants that are absent on host cells or because they lack “markers of self” that are normally present. CD47 (integrin-associated protein) functions are shown as a marker of self on murine RBCs. CD47 lacking RBCs were rapidly cleared from the bloodstream by splenic red pulp macrophages. CD47 receptor presence on RBCs prevented this elimination by binding to the inhibitory SIRPα where it induces phosphorylation, leading to the activation of protein phosphatase, which in turn inhibits the phagocytic synapses and eventually blocks phagocytosis^[117]^. Macrophages may use several nonspecific activating receptors and rely on the presence or absence of CD47 to distinguish themselves from foreign substances. Using the CD47 receptor to functionalize the NPs via cell membrane engineering or surface coating can help in longer circulation time and eventually better therapeutic outcomes in nanomedicine approaches. CD47 expressing cell membranes such as RBCs are co-extruded/coated along with other cell membranes to prepare cell membrane-coated NPs, which can avoid phagocytic elimination by macrophages and increase circulation time for prolonged drug release^[117]^.

#### *GPIb*α

GPIbα is a platelet membrane receptor and part of the glycoprotein family, and it mainly functions by facilitating adhesion onto von Willebrand factor (vWF) during vessel injury in the sub endothelium; this interaction between GPIbα and vWF is important for primary hemostasis and thrombus formation^[118]^. GPIbα forms a glycoprotein complex called GPIb-IX-V located on the surface of platelets composed of four leucine-rich glycoproteins including 135 kDa GPIbalpha with 626 amino acid length^[119]^. Blocking of this protein resulted in reduction of atherosclerosis in mice, which reveals its potential to be developed as a therapeutic approach for targeting this protein using anti-GPIbalpha-rich platelet membrane-coated NPs^[120]^. GPIbα was seen to be the prime mediator in non-alcoholic steatohepatitis, which progresses to hepatocellular carcinoma and treatment modalities involving antiplatelet therapies. Platelet cell membrane-coated NPs containing GPIbα or genetically overexpressed GPIbα can be employed for targeting platelet-derived cancer and tumor microenvironment responsive drug delivery for hepatocellular carcinoma^[120,121]^.

#### SNARE

soluble N-ethylmalemide-sensitive factor attachment protein receptors (SNARE) plays a major role in vesicle-mediated transport events. They are classified into v-SNAREs and t-SNARES, which help with the vesicle and target compartment, respectively^[122]^. V-SNARE has a coiled-coil homology domain with 60 amino acid length^[123,124]^. Various SNARE proteins, together, act to facilitate exocytosis and membrane fusion in cells, and SNAREs are also involved in the neurosecretion and orchestration of communicational aspects of neuronal and sensory cell synapses^[125]^. Membrane fusion is essential in delivery of intracellular proteins and improves efficiency of drug delivery to cells by aiding endosomal escape^[126]^. SNARE transmembrane domain is seen to be highly involved in the fusion-related functions of cell membranes^[127]^. Due to their fusogenic nature, investigating SNARE proteins for their use in functionalizing NPs for endosomal escape and improvement of cytosolic delivery is highly beneficial in drug delivery.

#### Mannose receptor

Mannose receptor (MR) or CD206 is a transmembrane receptor with a size of 175 kDa and a part of the mannose receptor superfamily. MR was reported to recognize and internalize specific groups of monosaccharides and lysosomal enzymes^[128]^. Mostly presented in DC, macrophages, and some nonvascular endothelial cells, MR plays an important role in the recognition and clearance of numerous endogenous antigens^[129]^. MR-mediated antigen capture is one of the two antigen capture mechanisms of DC^[130]^. It also aids in adaptive immunity by presenting the recognized antigen to the T cells for memory. Though the reasons are still unclear, MR was found to be present abundantly in gastric cancer and had a direct correlation with the severity of the cancer^[17]^. MR is also shown to play an immunoregulatory role in infectious diseases including those caused by HIV-1 and dengue virus. The virus enters the macrophages through the MR recognition of terminal mannose, fucose, or *N*-acetyl glucosamine moieties presented on the viral moieties. An interesting finding shows that MR mediates the retention of virions on the cell surface from detaching thereby downregulating the infection^[131,132]^. Based on the functional aspects associated with MR, it may serve as an essential antibody target for modulating immune responses, whether to induce or suppress immune responses by blocking its availability for pathogen entry. The potential of this receptor in cancer therapy is still unexplored, and the use of MR inhibitors or MR antibody-decorated NPs or cell membranes with MR expression coating drug NPs can be used to target gastric cancer or reduce viral titers by blocking MR and releasing antiviral drugs in a CMCNP-based approach.

#### CAR

Lymphocytes express different types of proteins on their membrane surface to detect diseased or inflamed tissues^[133,134]^. In addition, human lymphocytes express high levels of adhesion molecules to reach affected or activated tissue^[135,136]^ and are more effective in targeting tumor sites^[137,138]^. Therefore, the lymphocyte membrane on the surface of a drug carrier can increase specific tumor accumulation and target tissue interactions via native lymphocyte cell membrane adhesion molecules, which the tumor microenvironment also has in increased expression levels of the ligands for those adhesion molecules^[40]^. Some studies have reported improved targeting to tumor; for example, cytotoxic T lymphocyte- coated NPs have been used for targeting and treating gastric cancer^[46]^. Low-dose irradiation upregulated adhesion molecule expression in the tumor tissue, facilitating the increase in CD8+ T-cells in the tumor environment along with homing and localization of T lymphocyte membrane-encapsulated PLGA NPs. These T lymphocyte membrane-encapsulated NPs avoid opsonization via highly abundant serum proteins such as CD45 and CD3z and vascular extravasation by LFA-1 or CD11a, showing a decrease in overall uptake when compared to bare NPs. In addition, other studies reported that T lymphocyte membrane-encapsulated PLGA NPs were able to avoid being segregated by lysosomes and retained their lymphocyte coating on the NPs while trapped in the endolysosomal compartments^[58]^. Furthermore, integrating specific targeting moieties on the surface of lymphocytes by engineering the cell membrane with CAR is going to ensure its specific targeting to the affected tissues such as tumors.

Engineering for CAR-T cells is gaining interest due to their recent success in treating hematological malignancies by overcoming cytotoxic effects and improving therapeutic efficiencies in drug-resistant tumors; more recent advances in CAR-T engineering are discussed elsewhere by Huang *et al.*^[139]^. Crittenden *et al.*^[140]^ reported that tumor-associated carcinoembryonic antigen and CAR-engineered Jurkat cells were actively accumulated *in vivo* on liver cancer cells compared to naive Jurkat cells for cell-based viral therapeutic delivery. Studies show that integrating the tumor-specific targeting receptor to the surface of lymphocytes is going to increase the targeting of biomimetic drug carriers to the affected tissue by boosting the abovementioned advantages of T lymphocyte cell membranes. It is hypothesized that the synergistic effect of having specific targeting CARs on the T lymphocyte cell membranes will provide additional benefits to drug carriers in terms of more specific targeting. Therefore, the strategy of targeting the molecule (CAR, TCR, ScFv and so on) in engineering T lymphocyte cell membrane-coated drug delivery is a promising approach for the biomimetic drug delivery field to be explored further. In comparison, T cell receptors (TCRs) have site-specific affinity, especially against tumors, which can be harnessed along with engineered CARs to coat the nanocarriers to address the inter-and intra-heterogeneity of tumors. Recently, a similar approach was reported by Ma *et al.*^[141]^, where glypican-3 receptor specific to hepatocellular carcinoma was expressed on the T cell membrane to generate CAR-T cells, and engineered cell membranes were used to coat near-infrared dye (IR-780)-loaded mesoporous silica NPs^[141]^. CAR-T cell membrane-coated particles showed a higher accumulation and tumor reduction in the liver compared to only T cell membrane-coated NPs and uncoated plain NPs^[141]^. This study shows the ability of a CAR-T cell membrane as a potential therapeutic approach to treat solid tumors, where CAR-T-cell therapy approaches have faced challenges in overcoming barriers such as penetration and surviving an immunosuppressive environment in solid tumors. Furthermore, use of CAR-T membrane-coated nanocarriers may open a new way for individualized and tumor-specific targeting by the patient’s own ligand- and membrane-coated NP delivery.

#### Fc receptors

Fc receptors have been detected across different hematopoietic cells and are highly involved in antibody-dependent immune responses, which show Fc receptors as a potential target in the treatment of infectious diseases^[142]^. FcγRs are different types of receptors in the IgG superfamily, and they have a vital functional role in the activation of cytotoxic activity of FcγR-positive cells including NK cells, monocytes, macrophages and neutrophils via IgG monoclonal antibodies. Antitumor effects of mAbs at CD20 and HER2 are Fc-dependent as shown in Fc receptor-deficient nude mice^[143]^. FcγRs are involved in autoimmunity in a way where they impart hyper-responsiveness through interactions of FcγRs with circulating immune complexes; for instance, in FcγR (FcγRIIa) transgenic mice, Ab treatment inhibited collagen II-induced arthritis by binding to human FcγRIIa^[144]^.

For decades, Fc receptors have been investigated in terms of various diseases ranging from malaria to autoimmune disorders. Therefore, their physiology and role are well understood in most of the cases today. For example, monoclonal antibody therapies targeting activating Fc receptors are one of the promising cancer treatment strategies. Another potential application includes acute and chronic inflammation therapies. Nevertheless, Fc receptor biology has not been fully explored and utilized enough by researchers so far. If we consider Fc receptor biology from the aspect of cell- and cell membrane-based therapies, it has a vast potential to have various applications in drug delivery, imaging, and autoimmune regulatory treatments. For instance, tumor heterogenicity is a big hurdle for effective drug delivery and treatment of cancer. Targeting specific Fc receptor-decorated carriers can be envisioned as a personalized medicine by allowing a la carte ligand decoration on the surface of these drug carriers. Autoimmune diseases have complex disease initiation and progression; Fc-binding cell membrane receptors can modulate disease progression by aiding in the inhibition of excessive antibody and immune complexes in circulation. CMCNPs can be developed on the basis of this receptor knowledge to address the hyper-responsiveness issues by alleviating side effects of soluble protein-based therapeutics.

### Cellular membrane components for cancer therapy and immunomodulation

#### SCARF-1

scavenger receptor class F (SCARF1) is an 86-kDa type I transmembrane protein that contains a serine- and proline-rich cytoplasmic tail, a short transmembrane domain and epidermal growth factor-like domains on the extracellular region^[145]^. Functions of SCARF-1 are involved in low-density lipoprotein (LDL) binding [including acetylated (Ac)- and oxidized (Ox)-LDLs], apoptotic cell recognition in a C1q- and phosphatidylserine-dependent manner, and apoptotic cell clearance *in vitro* and *in vivo*^[145]^. Cells undergoing apoptosis present phosphatidylserine (PS), which is bound by SCARF-1 activated complement factor, C1q, leading to clearance of those apoptotic cells^[146]^. Impairment in the clearance of apoptotic cells leads to autoimmune diseases, e.g., systemic lupus erythematosus (SLE)^[145]^. Shedding light on the SCARF-1 pathway not only reveals its fundamental role in physiology, but also, this new SCARF-1 pathway can be used in new research directions for various diseases, including autoimmune disorders. As most of the cell surface receptors present SCARF-1, it has the potential to be utilized in cell/cell membrane-based applications, such as modulating SLE autoimmunity, delivering payloads to atherosclerotic lesions by targeting LDL, and apoptotic cell imaging *in vivo* via C1q-PS binding features.

#### TRAIL

TRAIL is a 281-amino acid, type II transmembrane protein that shares the TNF homology domain (THD), a conserved sequence of 150 residues, located at the extracellular carboxy terminal end of the molecules with other members of the TNF superfamily^[147]^. TRAIL is expressed in tissues such as the small intestine, colon, placenta, and most cells of the hematopoietic tissue^[148]^. TRAIL interacts with five different receptors: TRAIL receptor 1, TRAIL receptor 2, decoy receptor 1, decoy receptor 2, and osteoprotegerin (OPG). TRAIL receptor 1 [death receptor 4 (DR4)] and TRAIL receptor 2 [death receptor 5 (DR5)] contain a death domain, which upon TRAIL binding, causes apoptosis via various caspase activations^[149]^. Decoy receptors 1 (DcR1) and decoy receptor 2 (DcR2) express on the cell surface like DR4 and DR5. The tissue distribution of DcR2 is like DR4 and DR5, whereas DcR1 is only found in the heart, kidney, liver, lung, placenta, peripheral blood leukocytes, and spleen^[150]^. Overexpression of DcR1 and DcR2 protects from TRAIL-induced apoptosis^[125,151]^. DcR2 achieves this by activating NF-kB which is known to increase apoptosis^[150]^. The fifth receptor is OPG, a soluble protein with low affinity for TRAIL, but it has an unclear function.

The function of TRAIL is to control autoreactive immune cells and surveillance against tumor development and metastasis. TRAIL can induce apoptosis in cancer cells with little to no cytotoxicity against healthy cells^[152]^. However, there is reported hepatocyte toxicity caused by the exogenous tags (polyhistidine or Flag) on recombinant soluble human TRAIL (rhTRAIL)^[153]^. Despite the shortcomings of TRAIL as a lone therapeutic agent, researchers are combining TRAIL with cells and cell membranes. For example, a recent study of expressing TRAIL on human adipose-derived stem cells (hADSCs) and coating them with NPs was employed to effectively treat glioblastoma multiforme^[154]^. Future research will continue to develop strategies for overcoming TRAIL resistance in cancer through combining TRAIL with chemotherapy, immunotherapy, nanotechnology, or synthetic biology. Cell membrane-based research can use TRAIL in conjunction with different targeting proteins, drug-loaded NPs, and cell types including bacteria to treat drug-resistant tumors.

#### PD-L1

programmed death-ligand 1 (PD-L1) is a 40-kDa transmembrane protein that acts as a checkpoint that is operated as a negative regulator of T cells creating immune tolerance^[155]^. It is upregulated in tissues in response to IFN-γ and other inflammatory mediators^[156]^. Tumor cells overexpress PD-L1 as an adaptative mechanism to avoid an immune response^[157,158]^. PD-L1 inhibitors/antibodies have been used as an effective treatment against various cancer types, including melanoma and NSLC^[159,160]^. The suppression of PD-L1 and the PD-1 interaction has been shown to reduce malignancy in various clinical trials. One major problem with using PD-L1 inhibition therapy is its non-specificity, where overall suppression of PD-L1 increases the risk of autoimmune disorders. This, on the other hand, opens a new strategy for cell/cell-membrane based therapies where PD-L1-expressing membranes could be used for avoiding systemic clearance such as RES, and thereby increasing circulation time to improve therapeutic efficiency. Xu *et al.*^[161]^ showed that anti-PD-L1 mAb-coated polyethylene glycol-poly (ε-caprolactone) NPs (PEG-PCL NPs) loaded with docetaxel showed significant killing in PD-L1-transfected gastric tumor cell lines in comparison with the isotype IgG Ab control coating. Engineered T cell membrane-expressing PD-L1 antibodies can be employed to coat drug-loaded NPs for treating solid tumors by blocking PD-L1 interaction with PD-1, improving immune responses and providing efficient site-specific drug release of chemotherapeutic drugs^[162]^.

#### Major histocompatibility complex

Major histocompatibility complex (MHC) is a close locus of genes that code for the proteins present on most cell surfaces, which help in the detection of foreign peptides. There are two main groups of MHCs, class I and class II, of which the latter is present in immune cells alone unlike class I which is present in most cells^[163]^. The MHC group also has a class III, which codes for other proteins such as complement proteins, cytokines (chemical messengers), and enzymes^[163]^. The important function of the MHC is to bind pathogenic peptides and display them on the cell surface for T-cell recognition^[164]^. Each MHC complex has multiple alleles for the same gene, which makes it hard for the pathogen to evolve against the MHC mechanism. MHCs are found to be one of the major causes of autoimmune disorders, including type 1 diabetes, multiple sclerosis, ulcerative colitis (UC), and rheumatoid arthritis (RA), which arise when MHC class II presents self-antigens to autoreactive T lymphocytes due to loss of immunotolerance for some self-antigens^[165]^. MHCs play an important role in inflammatory regulation as seen in the study by Espel *et al.*^[166]^, where binding of TSST‐1 or LPS to MHC enhanced the transcriptional and translational rate of TNF‐α, a systemic inflammatory cytokine. Further research must be done on the pathways by which MHC molecules are linked to autoimmune diseases. This could open new directions for genetic engineering modalities against autoimmune diseases. Targeted therapies against specific MHCs could deliver drugs *in situ* against inflammation. Molecular biology techniques can also be employed to express specific antigen-presenting MHC molecules on cells and bacteria, and those engineered cell membranes could be used for coating NPs to be used as vaccines.

#### Bromodomain-containing protein 4

Bromodomain-containing protein 4 (BRD-4) is a human protein from the bromodomain family, which is largely known for its expression regulation properties of various oncogenes including Myc^[167,168]^. BRD-4 is expressed on cells of all tissues, and deregulation of BRD-4 has been shown to cause various diseases including cancer and fibrosis^[169]^. BRD-4 is shown to stabilize and help the growth of carcinoma and inhibition of BRD-4 using gene silencing or inhibitors, which significantly reduced tumor progression^[170]^. BRD-4 is also being studied as an effective regulator for fibrosis. Ding *et al.*^[171]^ showed that BRD-4 is a crucial component for the induction of profibrotic genes and aids in the activation of hepatic stellate cells (HSCs). It is shown that BRD-4 does not directly cause fibrosis, but rather aids in the induction of profibrotic factors including TGF-β, and that using BRD-4 inhibitors (JQ1 and I-BET) would downregulate the profibrotic effects^[172,173]^. The potential of BRD-4 as a biomarker and regulator for various diseases including fibrosis and cancer is promising. Mesenchymal stromal cells have great potential in the treatment of inflammatory diseases because of their affinity towards inflammatory signals. Engineering the Mesenchymal stromal cells to express BRD-4 inhibitors could act as a twofold mechanism treatment modality against fibrosis by inhibiting the induction of profibrotic factors and remodeling the inflammatory environment. BRD-4 gene silencing is also a field that is yet to be explored in the area of fibrosis.

## CONCLUSION AND FUTURE ASPECTS

Conventional synthetic payload delivery systems have a “foreign” material effect *in vivo* and some of the clinically approved materials today still cause immune system activation (complement or innate) and other toxic side effects to T cells owing to the mode of administration, material choice, and other pharmacodynamically varied profiles^[104]^. These challenges in conventional synthetic nanocarriers demand for a more advanced biomimetic drug delivery platform, where there is a balance between therapeutic activity and toxicity in healthy cells by drug carriers. Cancer drug resistance is caused by various factors including low bioavailability of drugs in deeper tumor sites, multiple drug resistance, tumor heterogeneity and other pharmacokinetic issues encountered *in vivo*. Cell- and cell membrane-based therapies can aid in overcoming cancer drug resistance by taking advantage of cell intrinsic properties of homing abilities with regard to targeting inflammation sites and infiltration into tumor regions. This potential opening avenues to engineer synthetic circuits producing therapeutic bioactive molecules *in situ* and other drug payload-carrying abilities, together, can improve drug bioavailability in the target location (e.g., tumor microenvironment) irrespective of cancer cell heterogeneity and other phagocytic issues faced by surface functionalized NPs.

**Figure 4.**
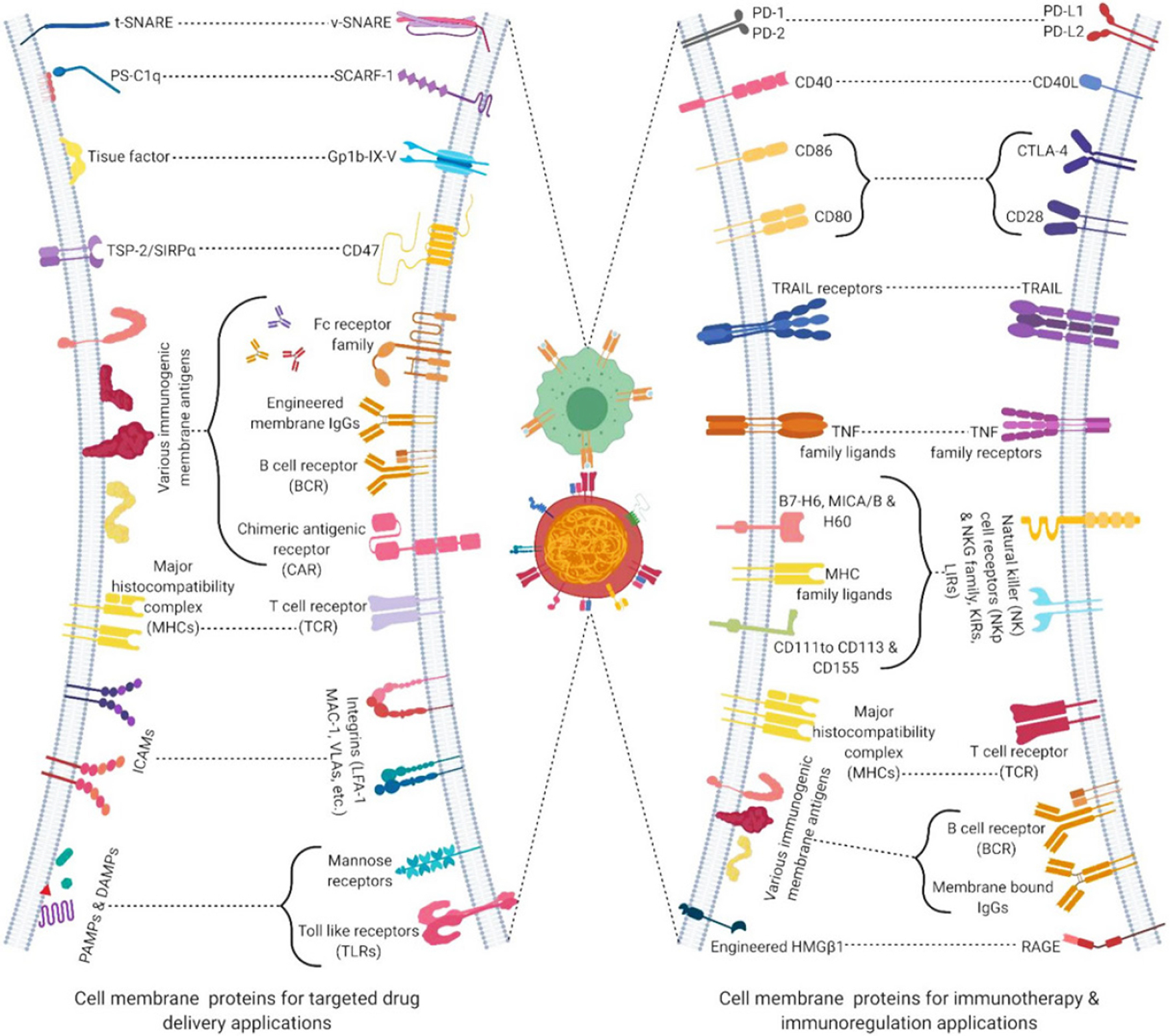
Potential cell surface proteins and their complements to be used in immunomodulation, immunotherapy, and targeted-drug delivery applications. t-SNARE/v-SNARE: target snap receptor/vesicle snap receptor; PS: phosphatidylserine; C1q: complement component 1q; SCARF-1: scavenger receptor class-F, member-1; Gp1b: glycoprotein-Ib; TSP-2: thrombospondin-2; SIRPα: signal regulatory protein α; CD: cluster of differentiation; ICAM: intercellular adhesion molecule; LFA-1: lymphocyte function-associated antigen-1; MAC-1: macrophage adhesion ligand-1; VLA: very late antigen; PAMP: pathogen associated molecular pattern; DAMP: damage-associated molecular pattern; PD-1/PD-2: programmed cell death protein-1/programmed cell death protein-2; PD-L1/PD-L2: programmed deathligand-1/programmed death-ligand-2; CTLA-4: cytotoxic t-lymphocyte-associated protein-4; TRAIL: tumor necrosis factor-related apoptosis-inducing ligand; TNF: tumor necrosis factor; B7-H6: B7 homolog 6; MIC: MHC class I polypeptide-related sequence; H60: histocompatibility protein-60; NKp: natural cytotoxicity triggering receptor; NKG: natural killer cell granule protein; KIR: killer-cell immunoglobulin-like receptor; LIR: leukocyte immunoglobulin-like receptor; HMGβ1: high-mobility group protein β1; RAGE: receptor for advanced glycation end products

Cell membrane-based therapeutic applications are mainly reported in the sense of passive-active targeted payload delivery, prolonged circulation and performing different types of therapeutic purposes on affected areas. Major advantages of CMCNPs include eliciting physiologically relevant immune responses, avoiding clearance while improving circulation and enhanced targeting via retained membrane proteins on NPs. All these properties together can improve the therapeutic efficacy of chemotherapeutic drugs in treating cancers. Various receptor-coated cell membranes can improve the abilities of synthetic NPs to facilitate combinatorial therapies, e.g., receptor-mediated apoptosis and immunomodulation, provision of fusion with cell membranes to escape endocytosis, in addition to NP-based chemotherapy in cancer. These multifunctional approaches lead to improved drug bioavailability at tumor sites, antigen presentation and immune cell maturation, improved adhesion via integrins, cell adhesion molecules and other potential receptor-mediated cancer therapeutics [Figure 4].

The cell membrane takes part in various critical biological tasks such as cell-cell signaling, inhibition or activation of cascades, and intrinsic and secretory pathways. Therefore, membrane-related proteins hold a very critical role in pathogenesis and progression of various acute and chronic diseases, including chronic infection, inflammation, cancer, autoimmune diseases, and other systemic disorders. Though the cell membrane coating of NPs can improve their physiochemical characteristics a great deal as discussed in the paper, it surely is not the end of the road. New research is focusing on new ways to improve membrane-coated NPs with pre- and post-membrane modification techniques^[174]^. Adding a targeting ligand to membrane-coated NPs has shown to greatly improve the targeting and retention properties of NPs without compromising the advantages of membrane coating. Fang *et al.*^[175]^ showed that lipid-assisted insertion of targeting aptamer showed very high targeting and uptake properties towards the cancer cells compared to plain membrane-coated and folate-conjugated membrane-coated NPs^[175]^. Various other researchers have also shown the increased functionality of lipid-assisted ligand insertion in membrane-coated NPs^[176,177]^. Cell membrane engineering techniques, both genetic (e.g., transfection) and other non-genetic engineering approaches (e.g., biotinylation, lipid membrane fusion, enzymatic or direct covalent, hydrophobic and electrostatic interactions), can be used to engineer cell membranes towards cell-based therapies including NP payload delivery against cancer^[178]^. Krishnamurthy *et al.*^[179]^ showed that transfecting proline, alanine and serine (PAS) expressing plasmid to cells and then using the membrane to coat NPs showed improved circulation with the increased targeting and retention property of membrane-coated NPs^[179]^. By taking advantage of proteomic techniques, one can identify pathologically significant membrane protein biomarkers and engineer therapeutically significant, patient-specific cell membrane components (e.g., CAR-T cells for the coating of nanocarriers) showing promise in personalized medicine^[180,181]^. Similarly, transfected cell membranes with various ligands and expressing genes could be used to coat NPs to produce synergic effects of the cell membrane with the transfected ligand/gene function.

The CMCNP core still utilizes synthetic materials for drug loading, which may pose potential side effects due to their accumulation *in situ* and elicit toxicity to the filtering organs such as kidneys and liver because of their extended elimination rates (e.g., longer retention time of metallic NPs *in vivo*)^[182]^. In that regard, research focused on preparation of biodegradable materials with improved pharmacokinetic and pharmacodynamic profiles for drug loading is highly desired. Overall, cell membrane-coated NP technology needs a multidisciplinary approach including the fields of membrane biology, bioengineering, molecular biology, proteomics, and pharmacology to develop more efficient cell- and/or cell membrane-based drug delivery systems with better safety and therapeutic efficacy in the treatment of cancers and immunomodulation-dependent diseases (e.g., autoimmune diseases).

## Figures and Tables

**Table 1.** Current applications of cell membrane-based nanoparticles in cancer therapies

Ligand	CMCNP type	Advantages	Application	Ref.
CD47	RBC membrane-coated PLGA NPs	Increased circulation half-life of NPs with an immunosuppressive CD47 marker towards SIRPα in phagocytic cells such as macrophages	Personalized medicine	[41,42]
CD47, CD235α, CD61 and CD41	RBC-platelet-coated PLGA NPs	CD235a marks a species-specific marker on the RBC along with CD47, an immunosuppressive marker, CD41 and CD61, making up the α_lb_β_3,_ assists in hemostasis and thrombosis of platelets	Personalized medicine	[43,44]
Cadherins and glycoprotein100	Mouse melanoma and cell membrane-coated PLGA NPs	Induction of dendritic cell maturation and stimulation of antigen-specific T cells by gp100 epitope	Cancer immunotherapy	[45]
CD45, CD3z and CD11a	Cytotoxic T lymphocyte membrane PLGA NPs	Ability to avoid opsonization via CD45 and CD3z markers and facilitate vascular extravasation via LFA-1 or CD11a	Cancer immunotherapy	[46]
α4 Integrin	Liposomes coated with macrophage membranes for targeting metastatic cancer cells	Macrophage α4 interactions with metastatic cancer cell VCAM-1 molecules to target and inhibit metastasis	Drug delivery for antimetastatic immunotherapy	[8]
Mac-1, N-cadherins	PLGA NPs coated with neutrophil membranes	Neutrophil membrane proteins such as Mac-1 and N-cadherin facilitate CTC-targeting properties and help design DDS for targeting metastatic niches	Antimetastatic immunotherapy	[11]
FGFRs, EGFRs	Stem cell membrane-coated nanoparticles	Stromal cell proliferation signal responsive receptors such as FGFRs and others on MSCs home them to the tumors, and this phenomenon is being used for tumor targeting	Targeted tumor therapy	[10]
FAKs, RHO	Cancer cell membrane-coated magnetic iron oxide NPs	Surface specific proteins such as integrins, FAKs and RHO proteins provide a homing ability of cancer cell membrane-coated NPs in tumor-self targeting	Targeted tumor therapy	[45,47]
Anti-CD19 synthetic notch receptor	T cell expression via SynNotch receptors	Antigen specific expression (input) of desired proteins at desired locations via T cells	Cancer immunotherapy	[30]
RTK-based and GPCRbased chimeric receptors	DCas9-SynR in HTLA / HEK293 cells	Combinatorial antigen inputs for site-specific cellular responses/delivery of therapeutic agents	Cancer immunotherapy	[31]
Neutrophil extracellular traps (NETs)	Neutrophil cell membrane coated nanoparticles	Natural binding of NETs to circulating tumor cells by selective adhesion is targeted to deliver therapeutic drugs inhibiting CTCs	Anti-metastatic therapy	[9,11]
MSR, MMR, VCAM-1	Macrophage membrane-coated nanoparticles	The macrophage membrane actively targets cancer by respective ligand adhesion and delivers the therapeutic drugs via nanoparticle release	Cancer immunotherapy	[48]
CXCR4 and CD44	Cancer cell and glioma cell membrane-coated nanoparticles	Disruption of cancer cell migration towards fibroblasts by internalization of NPs with CXCR4 and CD44	Antimetastatic therapies	[49]
MPLA	Cancer cell membrane, MPLA-functionalized PLGA NPs	Maturation of dendritic cells via MPLA functionalized cancer cell membrane	Antitumor therapy	[45]
PAMPs	Outer membrane vesicle of Salmonella	PAMPs bind to PRRs and stimulate innate immunity activities	Vaccine	[50]
HER-2-specific affibody	E. coli K-12 W3110 strain outer membrane vesicle (OMV)	HER-2-homing AffiHer-2 OMVs siRNA delivery to target cancer cells	Bacteria-mediated cancer immunotherapy	[51]
Engineered lipid A moiety	*Salmonella enterica* with lipid A modification for evading immune system	Tumor treatment of colon adenocarcinoma, metastatic murine carcinoma and B16Bl6 melanoma via engineered *S. enterica*	Bacteria-mediated antitumor therapy	[50]

**Table 2.** Major cell types and their applications in cancer and immunomodulatory therapies

Cell type	Disease treated	Strategy	Ref.
T cells	Autoimmunity and cancer	Direct delivery of immunomodulatory drugs via T cell surface-conjugated nanoparticles	[85]
	Prostate cancer	Maleimide-functionalized nanoparticles conjugated to effector T cells	[86]
	Circulating tumor cells	TRAIL-coated lymphocytes	[12]
	Gastric cancer	Cytotoxic T lymphocyte membrane-coated nanoparticles combined with low-dose irradiation	[46]
	Melanoma	Nanoporous silicon particles coated with LLV.	[58]
	Melanoma	Melanoma peptide MHC-specific TCR-expressing T cell membrane-coated PLGA nanoparticles	[15]
Macrophages	Breast cancer	Doxorubicin-loaded mesoporous silica nanocapsules camouflaged with macrophage cell membranes	[87]
	Bacterial infection	Mouse macrophage cell membranes and fusing them with PLGA cores	[88]
Dendritic cells	Melanoma, lung, and colon carcinoma	Exosomes derived from dendritic cells	[86]
	Breast cancer	Monocyte cell membrane shell- and doxorubicin-loaded PLGA core	[89]
Neutrophils	Glioma	Neutrophils loaded with paclitaxel carrying liposomes	[90]
	Circulating tumor cells	Coating neutrophil membranes on carfilzomib-loaded PLGA nanoparticles	[11]
Red blood cells	Lymphoma	Red blood cell membrane- and doxorubicin-loaded PLGA nanoparticles	[7]
	MRSA infection	RBC membrane functionalized with pore-forming α-hemolysin fused with the surface of PLGA	[91]
	Pore-forming toxins	Nanosponge made of RBC-coated PLGA	[54]
Platelets	Melanoma and breast cancer	Conjugated anti-programmed-death ligand 1 on the surface of platelets	[92]
	Breast cancer and prostate cancer	Engineered platelets to express membrane-bound TRAIL	[93]
	Myeloma	Bortezomib-loaded nanoparticles covered by alendronate-conjugated platelet membranes	[94]
	Circulating tumor cells	Designed silica nanoparticles coated by TRAIL-conjugated platelet membranes	[93]
Stem cells	Lung adenocarcinoma and ovarian cancer	Engineered human MSCs with paclitaxel-loaded polymeric nanoparticles	[95]
	Cervical cancer	Bone marrow derived mesenchymal stem cell membrane-coated gelatin nanogels loaded with doxorubicin	[10]
	Glioblastomas	Bone marrow derived MSCs loaded with paclitaxel encapsulated PLGA nanoparticles	[96]
Cancer cells	Breast cancer	Doxorubicin-loaded gold nanocages (AuNs) as an inner core and 4T1 cancer cell membranes (CMVs) as the outer shell	[97]
	Melanoma	CpG-loaded PLGA with B16-F10 mouse melanoma cell membranes	[23]
	Melanoma	MPLA modified mouse melanoma cancer cell membranes coated on PLGA nanoparticles	[45]
Bacteria	Carcinoma and melanoma	Lipopolysaccharide-inactivated *E. coli* outer membranes only	[98]
	HER-2 overexpressing tumors	Anti-HER-2 expressing *E. coli* membranes delivering small interfering RNA via targeting kinesin spindle protein	[99]
